# Gas-Phase Structures of Fucosylated Oligosaccharides: Alkali Metal and Halogen Influences

**DOI:** 10.1021/acs.jpcb.4c02696

**Published:** 2024-09-03

**Authors:** Samuel A. Miller, Kevin Jeanne Dit Fouque, Alexander M. Mebel, Kevin Brown Chandler, Francisco Fernandez-Lima

**Affiliations:** † Department of Chemistry and Biochemistry and Biomolecular Sciences Institute, Florida International University, 11200 SW Eighth Street, Miami, Florida 33199, United States; ‡ Translational Glycobiology Institute, Department of Translational Medicine, Herbert Wertheim College of Medicine, Florida International University, 11200 SW Eighth Street, Miami, Florida 33199, United States; § Biomolecular Sciences Institute, Florida International University, 11200 SW Eighth Street, Miami, Florida 33199, United States

## Abstract

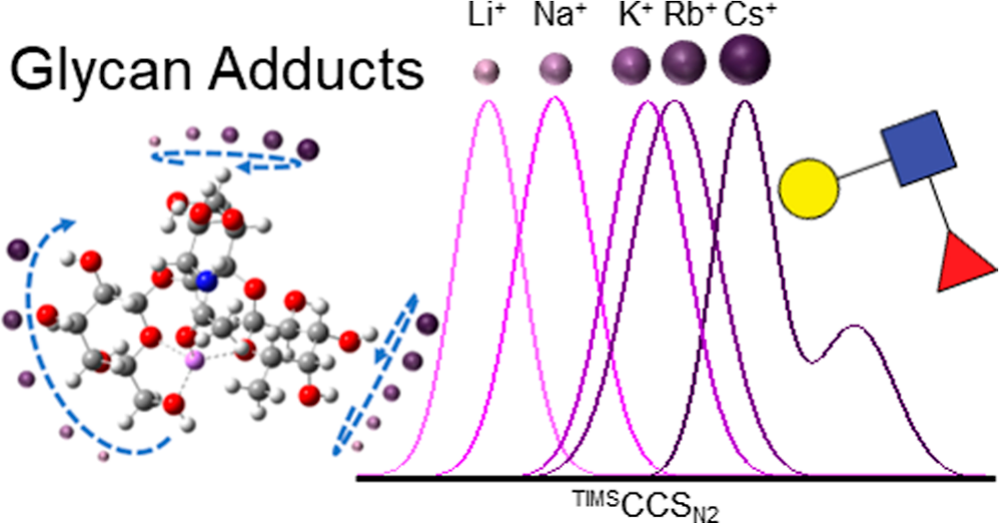

Fucosylated carbohydrate antigens play critical roles in physiology and pathology with function linked to their structural details. However, the separation and structural characterization of isomeric fucosylated epitopes remain challenging analytically. Here, we report for the first time the influence of alkali metal cations (Li^+^, Na^+^, K^+^, Rb^+^, and Cs^+^) and halogen anions (Cl^–^, Br^–^, and I^–^) on the gas-phase conformational landscapes of common fucosylated trisaccharides (Lewis A, X, and H types 1 and 2) and tetrasaccharides (Lewis B and Y) using trapped ion mobility spectrometry coupled to mass spectrometry and theoretical calculations. Inspection of the mobility profiles of individual standards showed a dependence on the number of mobility bands with the oligosaccharide and the alkali metal and halogen; collision cross sections are reported for all of the observed species. Results showed that trisaccharides (Lewis A, X, and H types 1 and 2) can be best mobility resolved in the positive mode using the [M + Li]^+^ molecular ion form (baseline resolution r ≈ 2.88 between Lewis X and A); tetrasaccharides can be best mobility resolved in the negative mode using the [M + I]^−^ molecular ion form (baseline separation *r* ≈ 1.35 between Lewis B and Y). The correlation between the number of oligosaccharide conformers as a function of the molecular ion adduct was studied using density functional theory. Theoretical calculations revealed that smaller cations can form more stable structures based on the number of coordinations, while larger cations induced greater oligosaccharide reorganizations; candidate structures are proposed to better understand the gas-phase oligosaccharide rearrangement trends. Inspection of the candidate structures suggests that the interplay between ion size/charge density and molecular structure dictated the conformational preferences and, consequently, the number of mobility bands and the mobility separation across isomers. This work provides a fundamental understanding of the gas-phase structural dynamics of fucosylated oligosaccharides and their interaction with alkali metals and halogens.

## Introduction

Fucosylated carbohydrate antigens are commonly found on glycoproteins and glycolipids, and play an outsized role in human physiology and pathology.^1^ Moreover, their biological function is tailored by their particular structural arrangement; for example, fucosylated carbohydrate isomers with fucose (Fuc) conjugated via the α(1,2), α(1,3), α(1,4), or α(1,6) linkage can be routinely observed.

The structural characterization and separation of isomeric fucosylated epitopes are analytically challenging. Previous efforts based on tandem mass spectrometry (MS/MS),^2^ diverse derivatization and fragmentation techniques,^3−5^ and/or chromatographic separation,^6−8^ including a recent method utilizing electrostatic repulsion hydrophilic interaction chromatography coupled with negative-ion electrospray MS/MS,^9^ are labor intense and lack specificity and sensitivity.

Metal ions attached to biomolecules play important roles in biological processes and their structural analyses have provided insights into the metal ion binding sites,^10,11^ coordination chemistry of metal–ligand interactions,^12,13^ and how metal interactions modulate structure and function relationships.^14,15^ Furthermore, the negative ion mode analysis of halogen anion bound species has gained traction compared to the previously stated traditional positive ion mode analysis providing complementary data for zwitterionic complexes or those in which elucidation/characterization is difficult.^16^ This allows for theoretical determination of molecular information regarding their energy, structure, and dynamics.^17,18^

Molecular characterization of complex mixtures using ion mobility spectrometry coupled to MS (IMS–MS) is rapidly becoming the analytical gold standard that combines the power of ultrahigh-resolution MS with the isomeric separation and structural identification capabilities of IMS.^19−22^ Moreover, the IMS measurement, when complemented with theoretical calculations, has proven to be a powerful technique for structural molecular analysis by correlating the ion-neutral collision cross sections (CCSs) with candidate structures. In the case of the separation of isomeric oligosaccharide species, IMS has shown some promises.^23−28^ Specifically, the potential of IMS for the analysis of polyaromatic hydrocarbons, explosives, metal porphyrins,^29,30^ carbohydrates,^31,32^ fucosylated human milk oligosaccharides,^33,34^ pharmaceuticals,^35,36^ and metabolites^37−39^ has been shown. While the addition of metal ion adducts can enhance the separation of isomeric species (e.g., carbohydrates,^40,41^ oligosaccharides,^26^ and peptides), little is known about the theoretical structure motifs and their impact on the folding/compaction of oligosaccharides.

Recent advancements in IMS techniques have significantly enhanced our ability to characterize complex carbohydrates with the use of metal adducts. Structures for lossless ion manipulations (SLIMs) and serpentine ultralong path with extended routing IMS have both demonstrated impressive ultrahigh resolution for glycans and lipids.^42−45^ Cyclic IMS (cIMS) and traveling wave IMS (TWIMS) have proven valuable in demonstrating their potential for discriminating epimeric glycans and glycopeptides as well as in the realm of metal adduction advancing carbohydrate sequencing challenges, while also exploring divalent cation adducts and their electron transfer products.^46−49^ While SLIM, TWIMS, and cIMS offer extended path lengths for enhanced separation, TIMS provides complementary benefits, particularly in its ability to trap and analyze ions, allowing for the detailed study of metal–glycan interactions.^50,51^ By comparing our results with those of these previous studies, we can highlight the unique insights provided by trapped ion mobility spectrometry coupled to mass spectrometry (TIMS–MS) in elucidating the structural dynamics of glycans under different ionic conditions, furthering our understanding of their gas-phase structures and intramolecular interactions.

**Figure 1:**
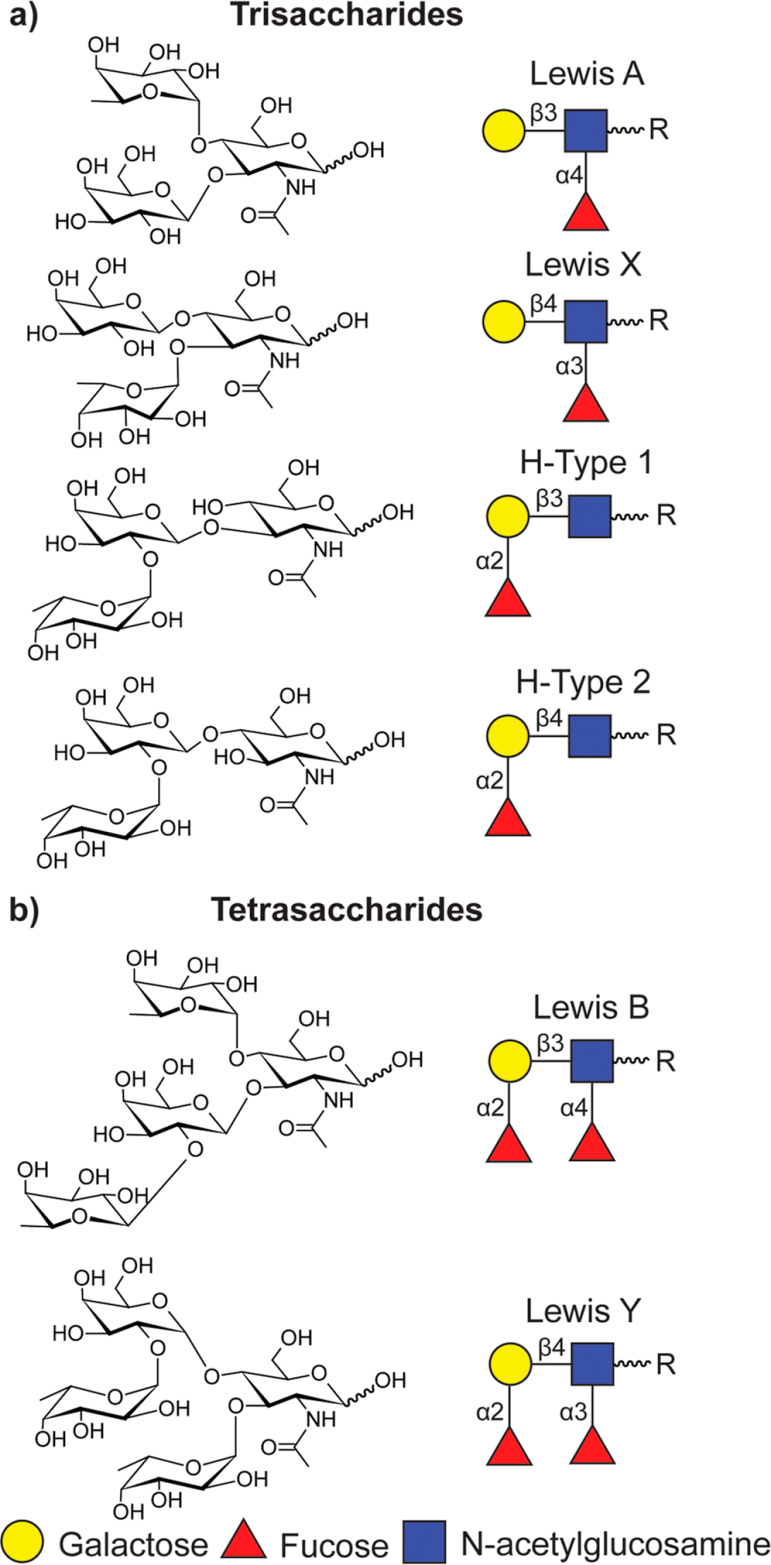
Chair conformations and schematic motifs for (a) trisaccharides Le^A^, Le^X^, BG-H^1^, and BG-H^2^ and (b) tetrasaccharides Le^B^ and Le^Y^. R = OH for oligosaccharides considered in this analysis.

In the present work, we report, for the first time, the influence of metal alkali (Li^+^, Na^+^, K^+^, Rb^+^, and Cs^+^) and halogen nonmetal ions (Cl^–^, Br^–^, and I^–^) on the gas-phase structures of commonly encountered fucosylated oligosaccharides: trisaccharides such as Lewis A (Le^A^), Lewis X (Le^X^), H antigen type 1 (BG-H^1^), and H antigen type 2 (BG-H^2^) (Figure 1a) and tetrasaccharides such as Lewis B (Le^B^) and Lewis Y (Le^Y^) (Figure 1b). Experimental IMS experiments showed the effect of the metal alkali and halogen ions on the conformational space and provided first-principles ion-neutral CCSs. Candidate structures are reported for all of the gas-phase conformational motifs observed using density functional theory (DFT) at the ωB97XD/6-31G(d) level.

## Methods

### Materials and Reagents

Oligosaccharides Lewis A {CAS: 56570–03–7; α-L-Fuc-(1 → 4)-[β-D-Gal-(1 → 3)]-D-GlcNAc], Lewis X {CAS: 71208-06-5; α-L-Fuc-(1 → 3)-[β-D-Gal-(1 → 4)]-D-GlcNAc}, H antigen type 1 [CAS: 137739-90-3; α-Fuc-(1 → 2)-β-Gal-(1 → 3)-GlcNAc], H antigen type 2 [CAS: 60797-31-1; α-Fuc-(1 → 2)-β-Gal-(1 → 4)-GlcNAc], Lewis B {α-Fuc(1 → 2)-β-Gal-(1 → 3)-(α-Fuc-[1 → 4])-GlcNAc}, and Lewis Y {α-Fuc-(1 → 2)-β-Gal-(1 → 4)-(α-Fuc-[1 → 3])-GlcNAc}, were purchased from Sigma-Aldrich (St. Louis, MO) as neat solids. Oligosaccharides were analyzed at a concentration of 10 μM in 50:50 methanol/water, which was fortified with a salt mixture containing lithium chloride (LiCl), sodium chloride (NaCl), potassium bromide (KBr), rubidium iodide, and cesium iodide to achieve a final concentration of 100 μM. Low concentration Tuning Mix standard (G1969–85000), obtained from Agilent Technologies (Santa Clara, CA) was used for the mobility and *m*/*z* calibration.^52,53^ The utilization of Agilent tune mix (containing HFAPs) for CCS calibration can have systematic subclass-dependent biases of +2–3% from reference drift tube CCS values.^54^ These biases may occur and be attributed to the structural mismatch between the phosphazine-based calibrants and the glycan analytes, similar to previous studies utilizing lipids.^54,55^

### IMS-MS Measurements

All experiments were performed on a custom built nanoESI-TIMS-*q*-ToF MS/MS (Bruker Daltonics Inc., Billerica, MA).^56^ Sample introduction via nanoESI was conducted by utilizing prepared in-house quartz capillaries (O.D. = 1.0 mm and I.D. = 0.70 mm) pulled via a Sutter Instrument Co. P2000 laser capillary puller (Novato, CA). Capillaries loaded with sample solution were mounted on a custom XYZ stage facing the instrument inlet, where voltages of ∼500–1200 V compared to the instrument source entrance was applied to induce electrospray ionization. A tungsten wire was inserted into the pulled nanoESI capillaries (Figure S.1). The dynamics and operation of the TIMS cell have previously been described.^57,58^ The TIMS–MS operation is controlled via custom in-house software within LabView (National Instruments, Austin, TX).

All TIMS–MS experiments were performed at an ambient temperature (*T*). Nitrogen (N_2_) was used for the ion mobility separation at a gas velocity (*v*_*g*_) defined by the pressure difference at the funnel entrance (*P*_1_ = 2.4 mbar) and exit (*P*_2_ = 0.9 mbar). An rf voltage of 250 *V*_pp_ at 880 kHz was applied to all electrodes. A voltage ramp (*V*_ramp_) of −200 to 0 V (positive ion mode)/190 to 40 V (negative ion mode), deflector voltage (*V*_def_) of 60 V (positive)/–60 V (negative) and base voltage (*V*_out_) of 60 V (positive)/–60 V (negative) were used to trap all species of interest. Higher resolution mobility separations utilized a narrower voltage ramp range Δ*V*_ramp_ = 25 V [trisaccharides *V*_ramp_ = −140 to −115 V (positive) and 135–110 V (negative) and tetrasaccharides *V*_ramp_ = −160 to −135 V (positive) and 160–135 V (negative)]. The scan rate (*S*_r_ = Δ*V*_ramp_/*t*_ramp_) was optimized to maximize the ion mobility separation in both positive and negative ion modes (0.05–2.0 V/ms) with *S*_*r*_ of 0.05 V/ms for both positive and negative modes.

The mobility resolving power (*R*) and resolution (*r*) were calculated utilizing their respective equations, *R* = Ω/*w* and *r* = 1.18 × (Ω_2_ – Ω_1_)/(*w*_1_ + *w*_2_), where Ω is the CCS of the analyte and *w* is the full peak width at half-maximum (fwhm) of the IMS band. Data were processed using Bruker Data Analysis 5.1 (Bruker Daltonics Inc., Billerica, MA), OriginPro 2021 (OriginLab Corp, Northampton, MA) graphing and analysis software, and GaussView 5.0 (Gaussian Inc., Wallingford, CT).

### Theoretical Section

A pool of candidate structures based on the stoichiometry of the observed molecular ions and adducts during the nESI-TIMS–MS experiments were proposed. The candidate structures adduct position and coordination was manually generated prior to geometry optimization and frequency calculation at the ωB97XD/6-31G(d) level of DFT.^59^ For Rb, Cs, and I atoms, the relativistic Stuttgart effective core potential^60^ was used to replace inner electrons, in conjunction with the accompanying basis set which is similar in quality to 6-31G(d). The Gaussian 16 package was employed in the DFT calculations.^61^ Zero-point vibrational energy corrections obtained from the calculations of vibrational frequencies were included in all energies of Me–OEP. Partial atomic charges were calculated using the Merz–Singh–Kollman scheme constrained to the molecular dipole moment.^62,63^ Theoretical mobility values were calculated using the trajectory method in nitrogen with Lennard-Jones (TMLJ) and added ion induced quadrupole (QPol) 4–6–12 potential (3,000,000 nitrogen molecules per rotation, three rotations, Maxwell distributed remission velocity, with a diffuse of zero, and zero accommodations assuming 100% elastic and specular collisions) in IMoS software (version 1.10cW64).^64−66^ Details on the optimized candidate geometries can be found in the Supporting Information. While the present isomeric search of possible complexes of tri- and tetrasaccharides with alkali cations and halogen anions was not exhaustive, it continued until the best match between the computed and experimentally measured ^TIMS^CCS_N_2__ was found.

## Results and Discussion

### (+) IMS-MS of Isomeric Trisaccharide Glycans

Positive ion mode IMS-MS analyses were performed on individual trisaccharides Lewis A (Le^A^), Lewis X (Le^X^), H antigen type 1 (BG-H^1^), and H antigen type 2 (BG-H^2^), as well as on a 1:1:1:1 mixture of the trisaccharides supplemented with 100 μM salt solution. All trisaccharides were observed in the single charge state as [M + Li]^+^ at *m*/*z* 536.2, [M + Na]^+^ at *m*/*z* 552.1, [M + K]^+^ at *m*/*z* 568.1, [M + Rb]^+^ at *m*/*z* 614.1, and [M + Cs]^+^ at *m*/*z* 662.1 when fortified with salt (Figure S.2).

**Figure 2:**
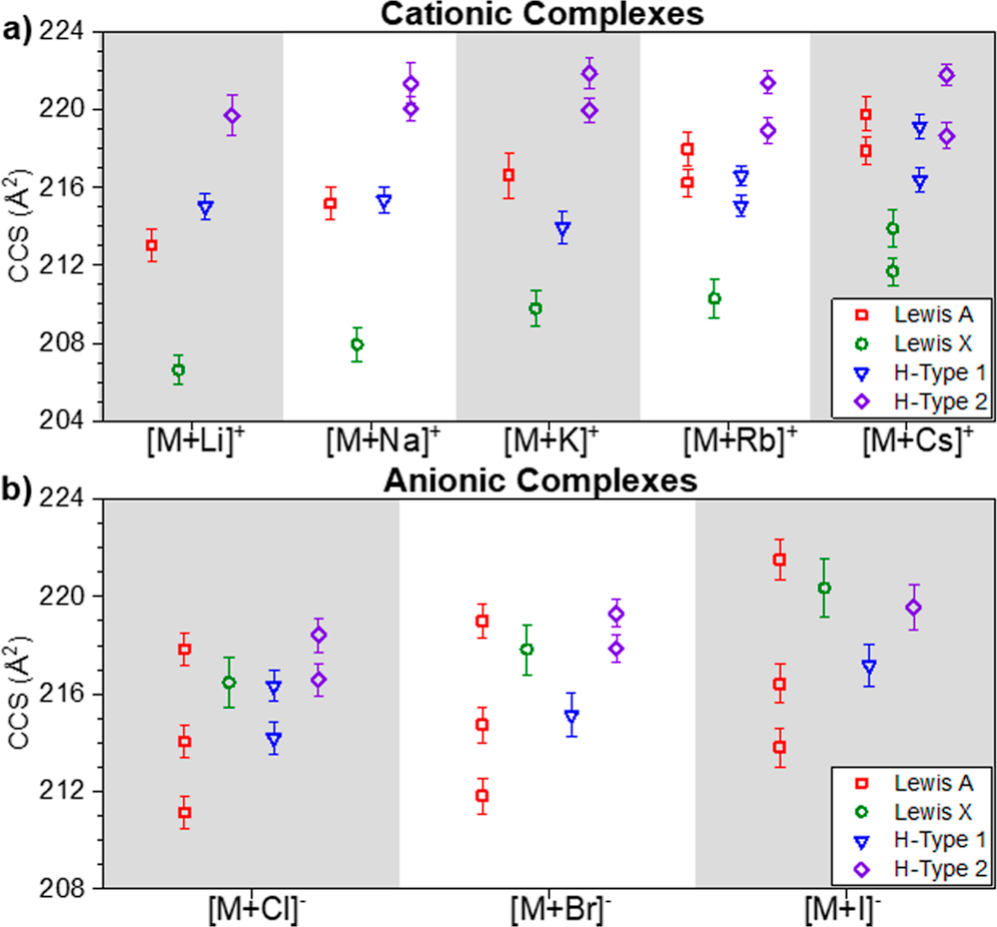
^TIMS^CCS_N_2__ values for trisaccharide glycans Le^X^ (green), Le^A^ (red), BG-H^1^ (blue), and BG-H^2^ (violet) as a function of the molecular ion adduct: (a) cationic and (b) anionic. Bars represent the IMS band fwhm using a Δ*V*_ramp_ = 25 V and *S*_r_ = 0.05 V/ms.

The [M + Li]^+^ molecular ion adducts showed single IMS bands per trisaccharide glycan with ^TIMS^CCS_N_2__ values of 206.6 (*R* ≈ 136.0), 213.0 (*R* ≈ 131.9), 215.0 (*R* ≈ 159.4), and 219.7 Å^2^ (*R* ≈ 103.6) for Le^X^, Le^A^, BG-H^1^, and BG-H^2^, respectively (Figure 2a). The [M + Na]^+^ molecular ion adducts of the isomeric trisaccharide glycan are only partially mobility resolved. The isomers showed a single IMS band with ^TIMS^CCS_N_2__ values of 207.9 (*R* ≈ 118.2), 215.2 (*R* ≈ 126.6), and 215.4 Å^2^ (*R* ≈ 159.6) for Le^X^, Le^A^, and BG-H^1^, respectively, and two IMS bands with ^TIMS^CCS_N_2__ of 220.1 (*R* ≈ 180.4) and 221.4 Å^2^ (*R* ≈ 105.7) for BG-H^2^. The [M + K]^+^ molecular ion adducts of the four isomeric trisaccharide glycans are mobility resolved. The isomer showed a single IMS band with ^TIMS^CCS_N_2__ values of 209.8 (*R* ≈ 113.5), 216.6 (*R* ≈ 92.0), and 214.0 Å^2^ (*R* ≈ 125.3) for Le^X^, Le^A^, and BG-H^1^, respectively, and two IMS bands with ^TIMS^CCS_N_2__ of 220.0 (*R* ≈ 170.6) and 221.9 Å^2^ (*R* ≈ 144.7) for BG-H^2^. The [M + Rb]^+^ molecular ion adducts of the isomeric trisaccharides glycan are only partially mobility resolved. The isomers showed a single IMS band with a ^TIMS^CCS_N_2__ value of 210.3 Å^2^ (*R* ≈ 103.5) for Le^X^ and two IMS bands with ^TIMS^CCS_N_2__ values of 216.2 Å^2^ (*R* ≈ 155.1) and 218.0 Å^2^ (*R* ≈ 130.4), 215.1 Å^2^ (*R* ≈ 189.2) and 216.6 Å^2^ (*R* ≈ 223.1), and 219.0 Å^2^ (*R* ≈ 168.5) and 221.4 Å^2^ (*R* ≈ 190.8) for Le^A^, BG-H^1^, and BG-H^2^, respectively. The [M + Cs]^+^ molecular ion adducts of the isomeric trisaccharides glycan are only partially mobility resolved. The isomers showed two IMS bands with ^TIMS^CCS_N_2__ values of 211.7 Å^2^ (*R* ≈ 147.6) and 213.9 Å^2^ (*R* ≈ 115.1), 217.9 Å^2^ (*R* ≈ 148.3) and 219.8 Å^2^ (*R* ≈ 125.5), 216.4 Å^2^ (*R* ≈ 165.9) and 219.1 Å^2^ (*R* ≈ 167.9), and 218.6 Å^2^ (*R* ≈ 163.4) and 221.8 Å^2^ (*R* ≈ 203.6) for Le^X^, Le^A^, BG-H^1^, and BG-H^2^, respectively.

The [M + Cl]^−^ molecular ion adducts of the isomeric trisaccharide glycan are only partially mobility resolved. The isomers showed a single IMS band for Le^X^ with ^TIMS^CCS_N_2__ of 216.5 Å^2^ (*R* ≈ 105.1), three IMS bands with ^TIMS^CCS_N_2__ values of 211.1 Å^2^ (*R* ≈ 129.0), 214.0 Å^2^ (*R* ≈ 132.4), and 218.0 Å^2^ (*R* ≈ 99.0) for Le^A^, and two IMS bands with ^TIMS^CCS_N_2__ values of 214.2 Å^2^ (*R* ≈ 151.4) and 216.4 Å^2^ (*R* ≈ 153.5) and 216.6 Å^2^ (*R* ≈ 162.7) and 218.4 Å^2^ (*R* ≈ 157.0) for BG-H^1^ and BG-H^2^, respectively (Figure 2b). The [M + Br]^−^ molecular ion adducts of the isomeric trisaccharides glycan are only partially mobility resolved. The isomers showed a single IMS band with ^TIMS^CCS_N_2__ of 217.8 Å^2^ (*R* ≈ 106.0) and 215.2 Å^2^ (*R* ≈ 115.1) for Le^X^ and BG-H^1^, respectively, three IMS bands with ^TIMS^CCS_N_2__ values of 211.8 Å^2^ (*R* ≈ 123.9), 214.7 Å^2^ (*R* ≈ 132.9), and 219.1 Å^2^ (*R* ≈ 127.6) for Le^A^, and two IMS bands with ^TIMS^CCS_N_2__ of 217.9 Å^2^ (*R* ≈ 151.4) and 219.5 Å^2^ (*R* ≈ 165.5) for BG-H^2^, respectively. The [M + I]^−^ molecular ion adducts of the isomeric trisaccharides glycan are only partially mobility resolved. The isomers showed three IMS bands with ^TIMS^CCS_N_2__ values of 213.9 Å^2^ (*R* ≈ 118.2), 216.4 Å^2^ (*R* ≈ 122.4), and 221.6 Å^2^ (*R* ≈ 107.8) for Le^A^, and single IMS band with ^TIMS^CCS_N_2__ of 220.2 Å^2^ (*R* ≈ 99.9), 217.2 Å^2^ (*R* ≈ 127.3), and 219.6 Å^2^ (*R* ≈ 119.9) for Le^X^, BG-H^1^, and BG-H^2^, respectively.

**Figure 3:**
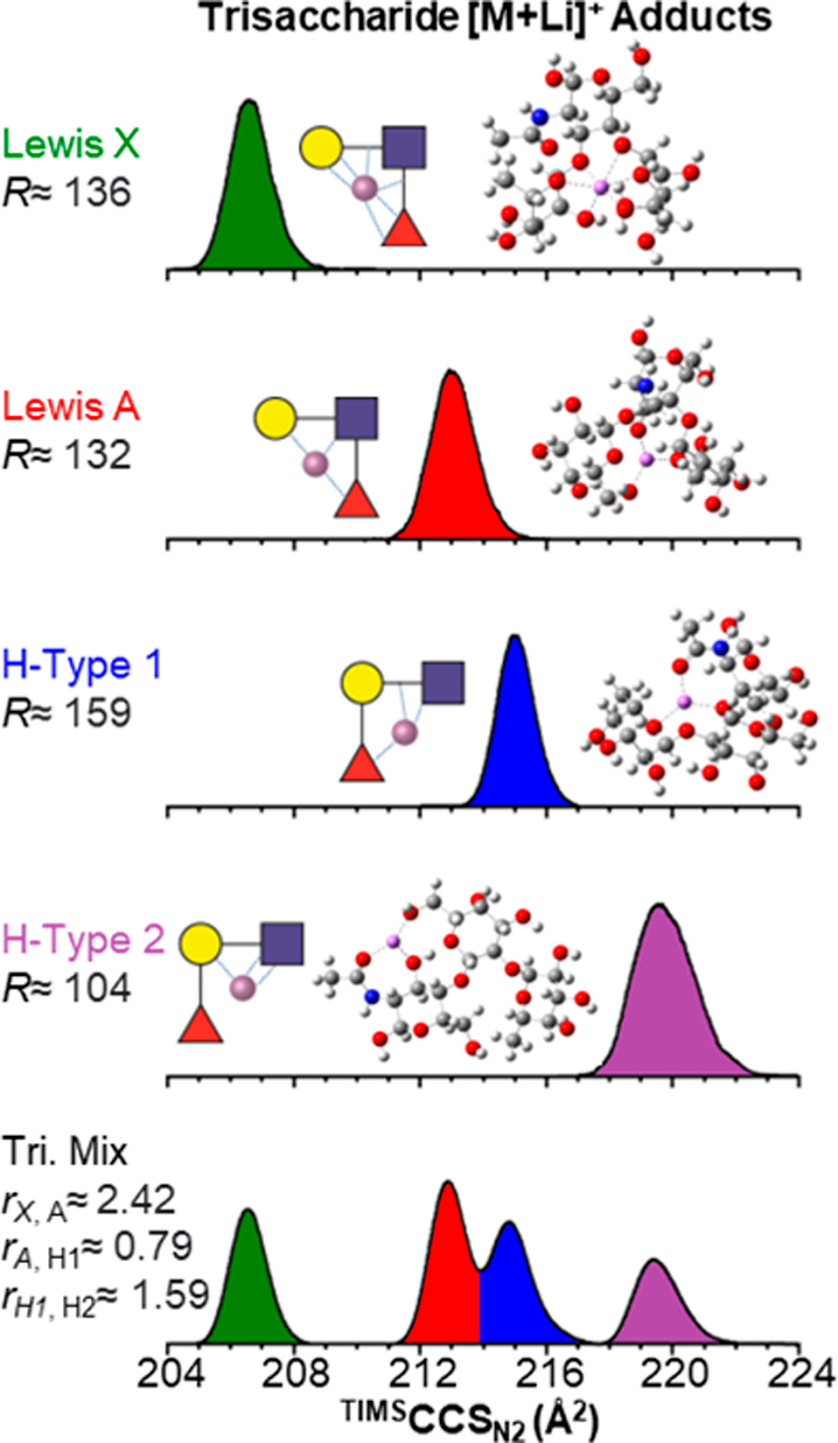
Typical ^TIMS^CCS_N_2__ profiles of the trisaccharide glycans Le^X^ (green), Le^A^ (red), BG-H^1^ (blue), and BG-H^2^ (violet) in the [M + Li]^+^ molecular ion adducts using Δ*V*_ramp_ = 25 V and *S*_r_ = 0.05 V/ms. In the insets, proposed coordination motifs and theoretical candidate structures are shown. (−) IMS-MS of isomeric trisaccharide glycans. Negative ion mode IMS-MS analyses were also performed on individual trisaccharides and on a 1:1:1:1 mixture of the trisaccharides supplemented with 100 μM of the salt solution. Three anionic single charge state adduct species, [M + Cl]^−^ at *m*/*z* 564.1, [M + Br]^−^ at *m*/*z* 608.1, and [M + I]^−^ at *m*/*z* 656.0, were observed in the mass spectra (Figure S.3).

Among all the considered alkali metal ions, the [M + Li]^+^ molecular ion adducts produced the best (+) mobility separation between the isomeric species (Figure 3) with baseline separation between [M + Li]^+^ Le^X^ and Le^A^ (*r* ≈ 2.88) and [M + Li]^+^ BG-H^1^ and BG-H^2^ (*r* ≈ 1.77), and near baseline between [M + Li]^+^ Le^A^ and BG-H^1^ (*r* ≈ 0.81). This effect is partially due to the simplicity of the mobility profiles, where mostly single IMS bands were observed, in contrast to other adducts where multiple bands were observed.

The optimized structures of the trisaccharide-Li^+^ adducts are illustrated in Figures 5 and S.39. The complexes with Le^X^, Le^A^, and BG-H^1^ trisaccharides, which have the lowest energies as compared to other probed isomers and give the best match with experimental ^TIMS^CCS_N_2__, feature coordination patterns, where the Li^+^ cation forms coordination bonds with all three saccharide monomers. These bonds are formed with oxygen atoms bearing a partial negative charge. In the complex with Le^A^, the three O atoms from three different monomers coordinated toward the metal cation include one from a hydroxyl group of the hydroxylmethyl (CH_2_OH) side chain of galactose (Gal), one from the carbonyl moiety of the acetamido (NHCOCH_3_) side chain of *N*-acetylglucosamine (GlcNAc), and one from the saccharide ring of fucose. A weaker coordination can be also seen toward the ring oxygen of galactose. In the Le^X^ complex, Li^+^ coordinates toward two OH oxygens from galactose and fucose and two O atoms from the ether moieties connecting GlcNAc with the other two monomers. In turn, in the BG-H^1^ complex, the coordination bonds of Li^+^ are formed with a hydroxyl oxygen of fucose, a carbonyl of the acetamido group in GlcNAc, and two ether oxygens in the connections of galactose with the other monomers. Only in the BG-H^2^ complex, the connection pattern differs and the Li^+^ cation coordinates to only two out of three saccharide monomers, the hydroxyl and carbonyl O atoms in GlcNAc and hydroxyl oxygen of hydroxylmethyl group in galactose. Another complex of BG-H^2^ which features a similar coordination pattern as in the BG-H^1^ complex was also found but it lies higher in energy (by 3.5 kcal/mol) and its ^TIMS^CCS_N_2__ does not match the experimentally observed value. The best (−) mobility separation was observed for the case of [M + Cl]^−^ molecular ion adducts, but due to the multiplicity of the species, only Le^A^ IMS1 (*r* ≈ 1.19) and BG-H^2^ IMS2 (*r* ≈ 0.65) can be differentiated, while remaining ion mobilities overlap.

### (+) IMS-MS of Isomeric Tetrasaccharide Glycans

Positive ion mode IMS-MS analyses were performed on the isomeric tetrasaccharide, Lewis B (Le^B^) and Lewis Y (Le^Y^), as well as 1:1 of the tetrasaccharide mixture supplemented with 100 μM salt solution. All tetrasaccharides were observed in the single charge state as [M + Li]^+^ at *m*/*z* 682.2, [M + Na]^+^ at *m*/*z* 698.2, [M + K]^+^ at *m*/*z* 714.1, [M + Rb]^+^ at *m*/*z* 760.1, and [M + Cs]^+^ at *m*/*z* 808.1 (Figure S.4).

The [M + Li]^+^ molecular ion adducts showed single IMS bands with ^TIMS^CCS_N_2__ of 236.7 Å^2^ (*R* ≈ 165.3) and 234.0 Å^2^ (*R* ≈ 170.0) for Le^B^ and Le^Y^, respectively (Figure 3). The [M + Na]^+^ molecular ion adducts showed two IMS bands with ^TIMS^CCS_N_2__ of 238.3 (*R* ≈ 211.3) and 239.9 Å^2^ (*R* ≈ 151.7) for Le^B^ and a single IMS bands with a ^TIMS^CCS_N_2__ of 235.2 Å^2^ (*R* ≈ 156.8) for Le^Y^. The [M + K]^+^ molecular ion adducts showed two IMS bands with ^TIMS^CCS_N_2__ values of 239.2 (*R* ≈ 170.7) and 241.4 (*R* ≈ 163.3) for Le^B^ and two IMS bands with ^TIMS^CCS_N_2__ values of 237.0 Å^2^ (*R* ≈ 150.2) and 239.4 Å^2^ (*R* ≈ 225.9) for Le^Y^. The [M + Rb]^+^ molecular ion adducts showed two IMS bands with ^TIMS^CCS_N_2__ values of 240.2 (*R* ≈ 184.7) and 241.9 (*R* ≈ 183.9) for Le^B^ and single IMS bands with a ^TIMS^CCS_N_2__ value of 237.9 Å^2^ (*R* ≈ 150.5) for Le^Y^. The [M + Cs]^+^ molecular ion adducts showed single IMS bands with ^TIMS^CCS_N_2__ values of 242.0 (*R* ≈ 139.4) and 240.0 Å^2^ (*R* ≈ 138.4) for Le^B^ and Le^Y^, respectively. In the first complex of Le^B^ with Na^+^, the cation is coordinated to the carbonyl group of GlcNAc and two hydroxyl oxygen atoms of the same Gal-bound fucose monomer (Figure S.39). This complex has the lowest energy and matches the lower, more intense ^TIMS^CCS_N_2__ peak. The second Le^B^ complex lies slightly higher in energy (by 0.9 kcal/mol) and has a higher ^TIMS^CCS_N_2__ value. The Na^+^ cation is coordinated toward the carbonyl oxygen of GlcNAc, the hydroxyl O of fucose, and the ether oxygen in the connection of the same fucose monomer with galactose. In the complex of Le^Y^, Na^+^ is linked with the carbonyl and ring oxygen atoms of GlcNAc.

Negative ion mode IMS-MS analyses were also performed on individual trisaccharides and on a 1:1 mixture of the trisaccharides supplemented with 100 μM salt solution. Three anionic single charge state adduct species, [M + Cl]^−^ at *m*/*z* 710.2, [M + Br]^−^ at *m*/*z* 754.1, and [M + I]^−^ at *m*/*z* 802.1, were observed in the mass spectra (Figure S.5).

The [M + Cl]^−^ molecular ion adducts showed two IMS bands with ^TIMS^CCS_N_2__ values of 236.4 (*R* ≈ 141.2) and 239.3 Å^2^ (*R* ≈ 154.6) for Le^B^ and a single IMS band with a ^TIMS^CCS_N_2__ value of 240.7 (*R* ≈ 117.5) for Le^Y^, respectively. The two [M + Cl]^−^ species are near-baseline separated (*r* ≈ 1.38) when comparing the dominant IMS bands for each species, and the Le^B^ second IMS band is less resolved (*r* ≈ 0.47). The [M + Br]^−^ molecular ion adducts showed two IMS bands with ^TIMS^CCS_N_2__ values of 237.2 (*R* ≈ 136.4) and 238.9 Å^2^ (*R* ≈ 182.7) for Le^B^ and a single IMS band with a CCS of 241.7 Å^2^ (*R* ≈ 110.3) for Le^Y^. The [M + I]^−^ adduct produced single IMS bands for each tetrasaccharide at ^TIMS^CCS_N_2__ values of 239.2 (*R* ≈ 135.2) and 243.5 (*R* ≈ 110.1) for Le^B^ and Le^Y^, respectively.

**Figure 4:**
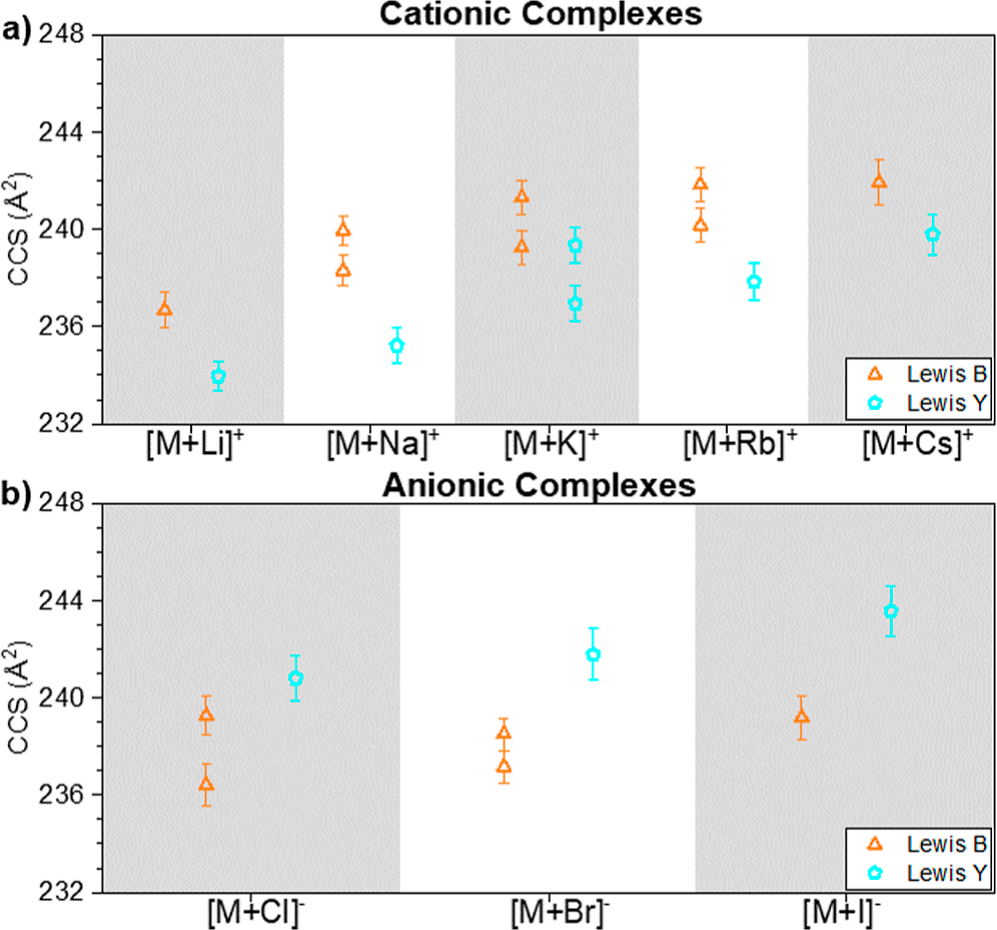
^TIMS^CCS_N_2__ values for tetrasaccharide glycans Le^B^ (orange) and Le^Y^ (cyan), as a function of the molecular ion adduct: (a) cationic and (b) anionic. Bars represent the IMS band fwhm using Δ*V*_ramp_ = 25 V and *S*_r_ = 0.05 V/ms.

Among all the considered adducts, the [M + Na]^+^ and [M + Cs]^+^ molecular ion adducts and the [M + Br]^−^ and [M + I]^−^ molecular ion adducts produced the best (+) and (−) mobility separation between the tetrasaccharide isomeric species, respectively. The [M + Na]^+^ and [M + Cs]^+^ tetrasaccharides are baseline resolved (*r* ≈ 1.38 and *r* ≈ 0.69, respectively) (Figure 4). The [M + Br]^−^ and [M + I]^−^ species are baseline separated (*r* ≈ 1.35 and *r* ≈ 0.96), respectively.

**Figure 5:**
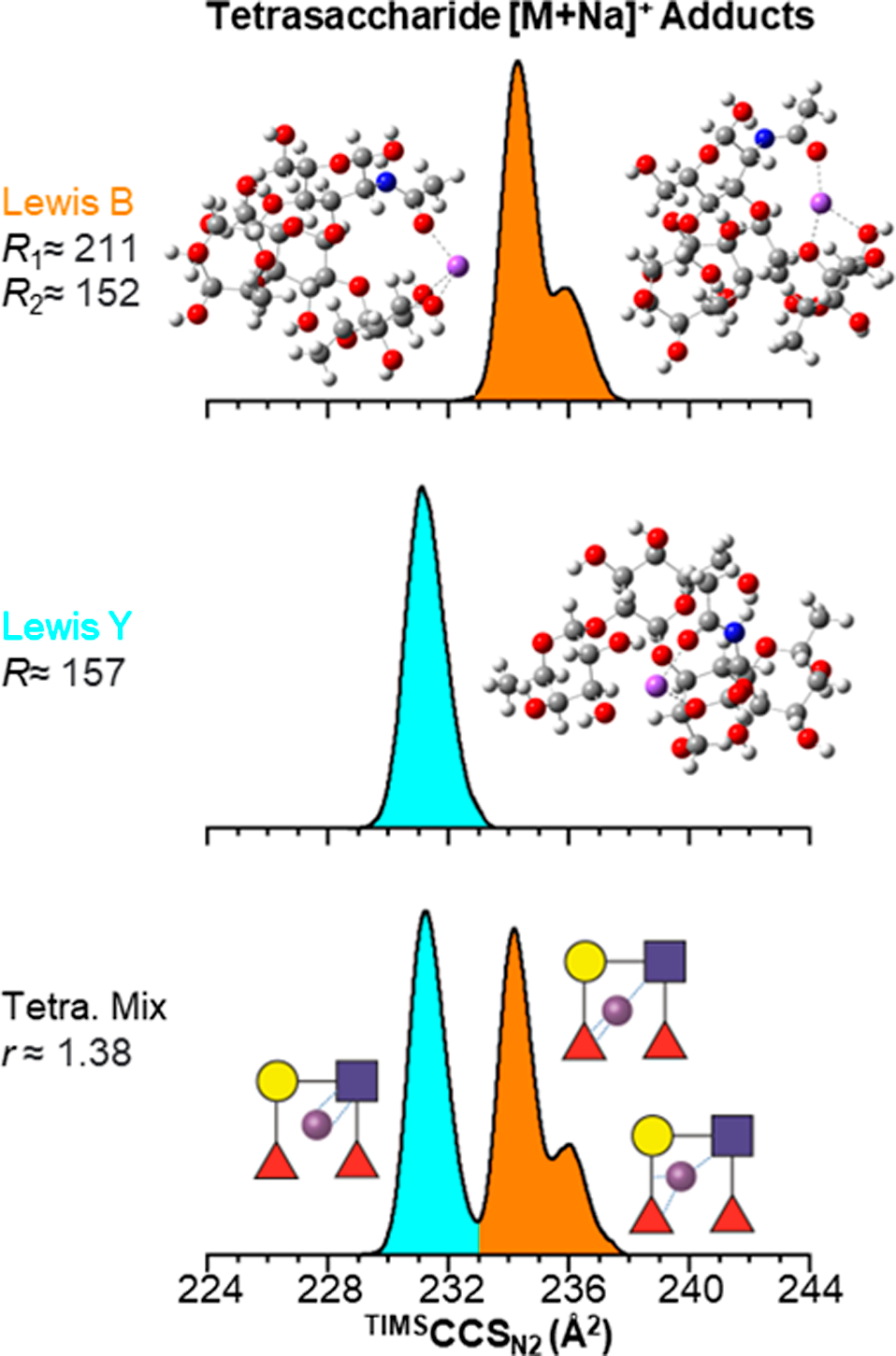
Typical ^TIMS^CCS_N_2__ profiles of the tetrasaccharide glycans Le^B^ (orange) and Le^Y^ (cyan) [M + Na]^+^ molecular ion adducts using Δ*V*_ramp_ = 25 V and *S*_r_ = 0.05 V/ms. In the insets, proposed coordination motifs and theoretical candidate structures are shown. (−)IMS-MS of isomeric tetrasaccharide glycans.

Separation of isomeric fucosylated oligosaccharides was achieved for the isomer quartet of trisaccharides as well as the pair of tetrasaccharides while operating in the positive ion mode. Greater separation was observed with smaller cations (Li^+^ and Na^+^) compared to larger ones (K^+^, Rb^+^, and Cs^+^). An increase in the CCS was observed with the size of the metal cation increased (Figure 5).

**Figure 6:**
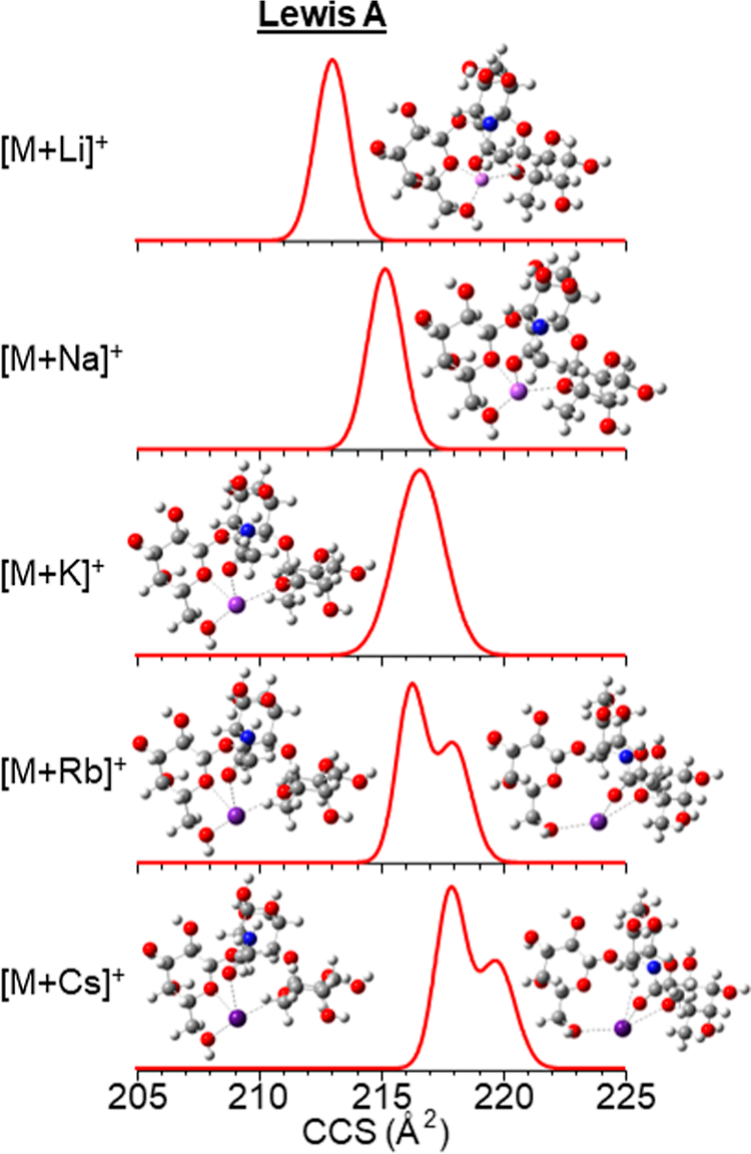
^TIMS^CCS_N_2__ ion mobility spectra (*S*_r_ = 0.05 V/ms) of oligosaccharide Lewis A and closest matching ^TIMS^CCS_N_2__ 3D theoretical structures of alkali metal cation species.

We interpret this trend as an effect produced to the increase in the atomic radius of the metal cation. As the metal ion adduct becomes larger, it occupies more space and can differentially interact with the surrounding molecular groups. The atomic radius increase also showed dependence on the number of conformers observed (Figure 6). For example, larger metal cation adducts produced more IMS bands compared to smaller ones due to their size and binding properties. These intramolecular constraints are driven by the saccharides since all of the metal ions had the same charge.

The ionic radius of a metal cation can influence its coordination number. There is no general rule in coordination chemistry dictating the relationship between the size of a metal cation coordination number due to multiple factors influencing it, including ligand size, metal-ion charge, solvent, and overall geometry. Nevertheless, smaller glycan–metal complexes generally have higher coordination numbers, due to their higher charge density, compared to larger metal cations, causing them to fit into the coordination sites of the glycans more effectively, allowing for stronger binding interactions and more stable complexes. The larger metal cations have difficulty fitting into these same sites due to steric hindrance, limiting them to lower coordination numbers and resulting in weaker binding interactions.

**Figure 7:**
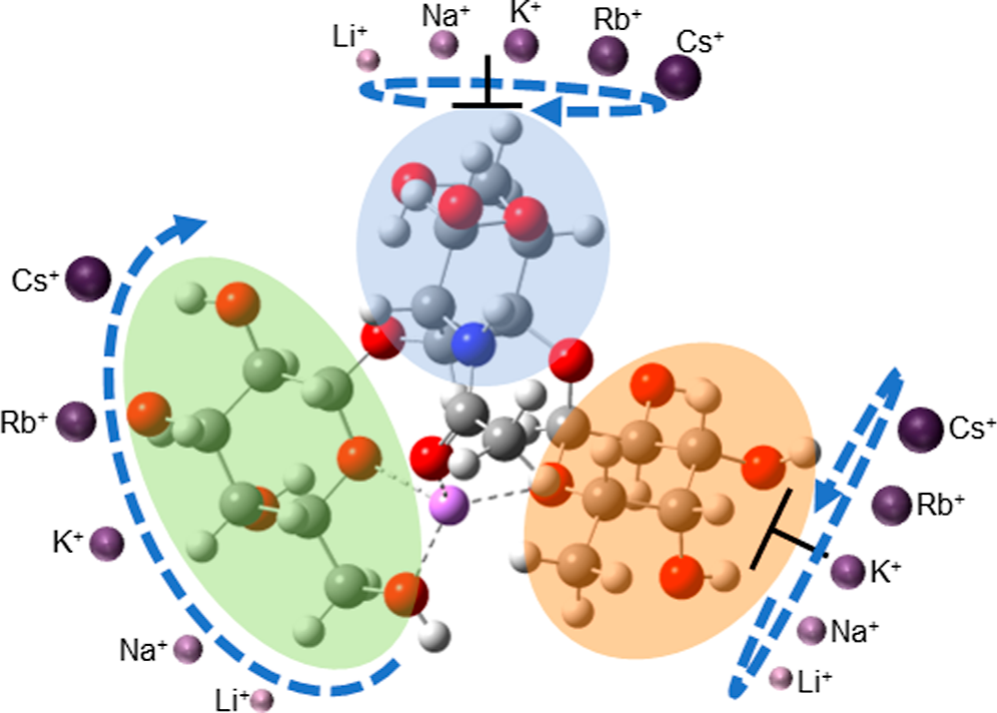
Simplified schematics of the effect of the alkali metal on the Lewis A conformational dynamics. A rotation and movement of the fucose (green), *N*-acetylglucosamine (blue), and galactose (orange) sugars is proposed with the increase in alkali metal size.

Theoretical calculations showed that these larger metals caused structural reformation of the sugars within the polysaccharides to accommodate the cations’ larger size within the binding site (Figure 7). As the cation size increases, the sugars continue to move in a consistent way. In addition, the theoretical modeling demonstrated multiple coordinations for the Le^A^ cationic species and for the lithiated and sodiated species of the isomeric trisaccharides and tetrasaccharides, in Figures 6 and S.39, respectively.

This is consistent with previous models performed by Zheng et al.^26^ suggesting that the sodium ion binds different locations on monosaccharide rings, causing changes in structural size. Different interactions/locations of Na^+^ (and other cations) with the oligosaccharides effectively contribute to differences in mobility separation based on cation size, as well as the production of multiple local free energy minima corresponding to different conformers. This is in contrast to previous findings based on the study of isomeric fucosylated tetrasaccharides lacto-*N*-tetraose (LNT) and lacto-*N*-neotetraose (LNnT), suggesting that fucose linkage likely also contributes to their separation.^67^

The presence of anomeric glycan species has been suspected to contribute to the observation of multiple ion mobility bands when analyzing metal and halogen adducts of glycans using various IMS platforms.^42,46,47,68,69^ This complexity can arise due to several factors: different anomeric forms (α and β) of glycans have distinct three-dimensional structures, leading to varying ion mobilities; metal and halogen coordination occurs at different sites depending on the anomeric configuration, resulting in diverse gas-phase structures; and the conformational flexibility of anomeric glycans, influenced by metal or halogen adduction, produces multiple stable conformers, each with distinct ion mobilities. Additionally, glycans in solution exist in equilibrium between anomeric forms, and this equilibrium may be “frozen” upon electrospray ionization, leading to the detection of multiple gas-phase species.^70,71^ These factors collectively can result in intricate mobility spectra, offering valuable structural insights but also posing challenges for data interpretation and assignment.

## Conclusions

This study described the intricate relationship between alkali metal cations and halogen nonmetal anions on the conformational space of isomeric fucosylated oligosaccharides and their corresponding analytical mobility separation observed using (±) ESI-TIMS-MS. In the positive ion mode, it was shown that smaller cations, such as Li^+^ and Na^+^, demonstrated greater separation efficiency compared to larger cations, resulting in baseline mobility separation of isomeric species. The coordination chemistry of metal cations defined the conformational space of the glycan–metal complex, with smaller cations forming more stable complexes due to higher coordination numbers (i.e., single IMS band), while larger cations experienced steric hindrance, leading to weaker bonding interactions and larger structural variability of the oligosaccharide–metal complex (i.e., multiple IMS bands). In the negative ion mode, mobility separations were achieved for all anionic species with iodated adducts exhibiting the highest resolution of the isomeric species. Overall, our findings underscore the significance of ion size and charge density in dictating the conformational space, leading to better analytical mobility separation as well as the anomeric influence on the mobility profiles of fucosylated oligosaccharides. This study provides valuable insights into the structural dynamics of glycan–metal and glycan–halide complexes, further advancing our understanding of the gas-phase intramolecular interactions that stabilize molecular structures.

## Supporting Information Available

3D xyz coordinates for all the candidate structures discussed (XLSX)
Single standard MS and MS/MS profiles and low- and high-resolution IMS profiles (PDF)
