# Supplementary material for: Gas-Phase Structures of Fucosylated Oligosaccharides: Alkali Metal and Halogen Influences
Source: J Phys Chem B. 2024 Sep 3;128(37):8869–77. doi: 10.1021/acs.jpcb.4c02696 (PMC11421426; doi:10.1021/acs.jpcb.4c02696)
Supplement: Supplementary file 2 — jp4c02696_si_002.pdf [file jp4c02696_si_002.pdf]

Gas-Phase Structures of Fucosylated Oligosaccharides: Alkali Metal and Halogen Influences

Samuel A. Miller[1], Kevin Jeanne Dit Fouque[1], Alexander M. Mebel[1], Kevin Brown Chandler[2,3,]*and Francisco Fernandez-Lima[1,]*

[1] Department of Chemistry and Biochemistry and Biomolecular Sciences Institute, Florida International University, 11200 SW 8th St., Miami, FL 33199, United States.

[2] Translational Glycobiology Institute, Department of Translational Medicine, Herbert Wertheim College of Medicine, Florida International University, 11200 SW 8th St., Miami, FL 33199, United States.

[3] Biomolecular Sciences Institute, Florida International University, 11200 SW 8th St., Miami, FL 33199, USA

SUPPLEMENTAL INFORMATION

Contents

Figure S.1: General schematic of the TIMS-q-CID-ToF MS instrumentation. S3

Figure S.2: Mass spectrum of isomeric trisaccharide mix.. .... S4

Figure S.3: Mass spectrum of isomeric trisaccharide mix.. .... S5

Figure S.4: Mass spectrum of isomeric tetrasaccharide mix... S6

Figure S.5: Mass spectrum of isomeric tetrasaccharice mix.. .... S7

Figure S.6: Normalized low resolution ion mobility spectra for the trisaccharide $[M+Li]^+$ cation. S8

Figure S.7: Normalized high resolutionion mobility spectra for the trisaccharide $[M+Li]^+$ cation. S8

Figure S.8: Normalized low resolution ion mobility spectra for the trisaccharide $[M+Na]^+$ cation. S9

Figure S.9: Normalized high resolution ion mobility spectra for the trisaccharide $[M+Na]^+$ cation S9

Figure S.10: Normalized low resolution ion mobility spectra for the trisaccharide $[M+K]^+$ cation. S10

Figure S.11: Normalized high resolution ion mobility spectra for the trisaccharide $[M+K]^+$ cation. S10

Figure S.12: Normalized low resolution ion mobility spectra for the trisaccharide $[M+Rb]^+$ cation. S11

Figure S.13: Normalized high resolution ion mobility spectra for the trisaccharide $[M+Rb]^+$ cation. S11

Figure S.14: Normalized low resolution ion mobility spectra for the trisaccharide $[M+Cs]^+$ cation. S12

Figure S.15: Normalized high resolution ion mobility spectra for the trisaccharide $[M+Cs]^+$ cation. S12

Figure S.16: Normalized low resolution ion mobility spectra for the tetrasaccharide $[M+Li]^+$ cation.... S13

Figure S.17: Normalized high resolution ion mobility spectra for the tetrasaccharide $[M+Li]^+$ cation. . S13

Figure S.18: Normalized low resolution ion mobility spectra for the tetrasaccharide $[M+Na]^+$ cation .. S14

Figure S.19: Normalized high resolution ion mobility spectra for the tetrasaccharide $[M+Na]^+$ cation . S14

Figure S.20: Normalized low resolution ion mobility spectra for the tetrasaccharide $[M+K]^+$ cation S15

Figure S.21: Normalized high resolution ion mobility spectra for the tetrasaccharide $[M+K]^+$ cation ... S15

Figure S.22: Normalized low resolution ion mobility spectra for the tetrasaccharide $[M+Rb]^+$ cation. . S16

Figure S.23: Normalized high resolution ion mobility spectra for the tetrasaccharide $[M+Rb]^+$ cation. S16

Figure S.24: Normalized low resolution ion mobility spectra for the tetrasaccharide $[M+Cs]^+$ cation... S17
Figure S.25: Normalized high resolutionion mobility spectra for the tetrasaccharide $[M+Cs]^+$ cation... S17
Figure S.26: Normalized low resolutionion mobility spectra for the trisaccharide $[M+Cl]^-$ anion S18
Figure S.27: Normalized high resolution ion mobility spectra for the trisaccharide $[M+Cl]^-$ anion S18
Figure S.28: Normalized low resolution ion mobility spectra for the trisaccharide $[M+Br]^-$ anion........ S19
Figure S.29: Normalized high resolutionion mobility spectra for the trisaccharide $[M+Br]^-$ anion........ S19
Figure S.30: Normalized low resolution ion mobility spectra for the trisaccharide $[M+I]^-$ anion........... S20
Figure S.31: Normalized high resolution ion mobility spectra for the trisaccharide $[M+I]^-$ anion.......... S20
Figure S.32: Normalized low resolution ion mobility spectra for the tetrasaccharide $[M+Cl]^-$ anion..... S21
Figure S.33: Normalized high resolution ion mobility spectra for the tetrasaccharide $[M+Cl]^-$ anion.... S21
Figure S.34: Normalized low resolution ion mobility spectra for the tetrasaccharide $[M+Br]^-$ anion..... S22
Figure S.35: Normalized high resolution ion mobility spectra for the tetrasaccharide $[M+Br]^-$ anion.... S22
Figure S.36: Normalized low resolution ion mobility spectra for the tetrasaccharide $[M+I]^-$ anion S23
Figure S.37: Normalized high resolution ion mobility spectra for the tetrasaccharide $[M+I]^-$ anion S23
Figure S.38: Ion mobility spectra of oligosaccharide Lewis A, coordination motifs, and 3D theoretical structures of halogen anion species... S24
Figure S.39: 3D theoretical candidate structures with coordination site bond lengths trisaccharide $[M+Li]^+$ adducts, tetrasaccharide $[M+Na]^+$ adducts, and Le^A bound with all cation adducts.................. S25
Table S.1: Summary of experimental positive and negative mode $^{TIMS}CCS_{N2}$ and m/z values of IMS adducts compared to theoretical model CCS values. S26
Table S.2: Summary of experimental positive and negative mode $^{TIMS}CCS_{N2}$ and *m/z* values of IMS adducts with calculated resolving power (*R)* and resolution (*r*) values. S28
Figure S.40: MS2 (CID) Spectrum - Lewis A Trisaccharide $[M + Na]^+$.. S30
Table S.3: Observed Fragments in MS2 (CID) Spectrum - Lewis A Trisaccharide $[M + Na]^+$.............. S31
Figure S.41: MS2 (CID) Spectrum - H-type 2 Band 1 $[M + Na]^+$... S32
Table S.4: Observed Fragments in MS2 (CID) Spectrum H Antigen Type 2 $[M + Na]^+$ Band 1............ S33
Figure S.42: MS2 (CID) Spectrum - H-type 2 Band 2 $[M + Na]^+$... S34
Table S.5: Observed Fragments in MS2 (CID) Spectrum H Antigen Type 2 [M + Na]+ Band 2........... S35
Figure S.43: MS2 (CID) Spectrum - Lewis Y Tetrasaccharide [M + Na]+.. S36
Table S.6: Observed Fragments in MS2 (CID) Spectrum Lewis Y [M + Na]+...................................... S37
Figure S.44: MS2 (CID) Spectrum - Lewis B [M + Na]+ IMS 1 .... S38
Table S.7: Observed Fragments in MS2 (CID) Spectrum Lewis B [M + Na]+ IMS 1............................ S39
Figure S.45: MS2 (CID) Spectrum - Lewis B Band 2 [M + Na]+.. S40
Table S.8: Observed Fragments in MS2 (CID) Spectrum Lewis B [M + Na]+ IMS 2............................ S41

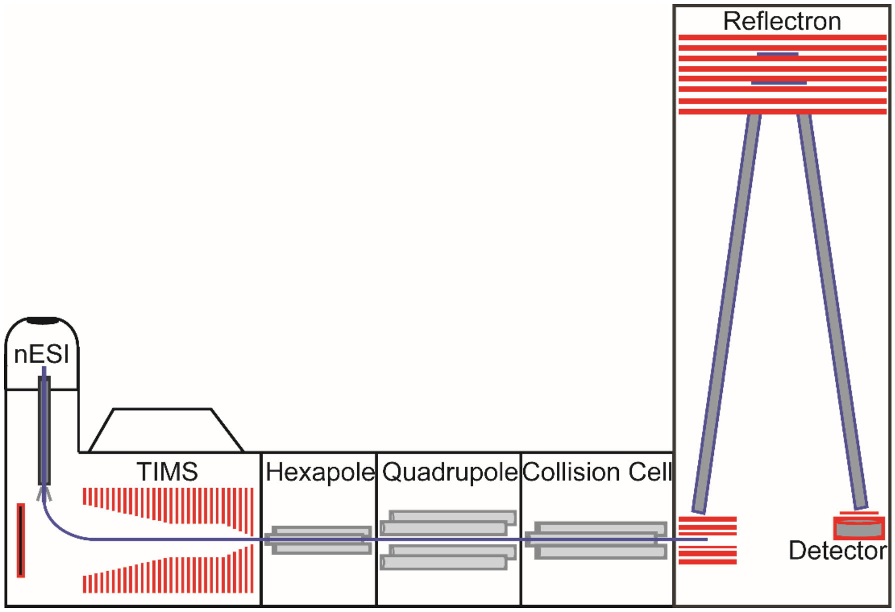

Figure S.1: General schematic of the TIMS-q-CID-ToF MS instrumentation.

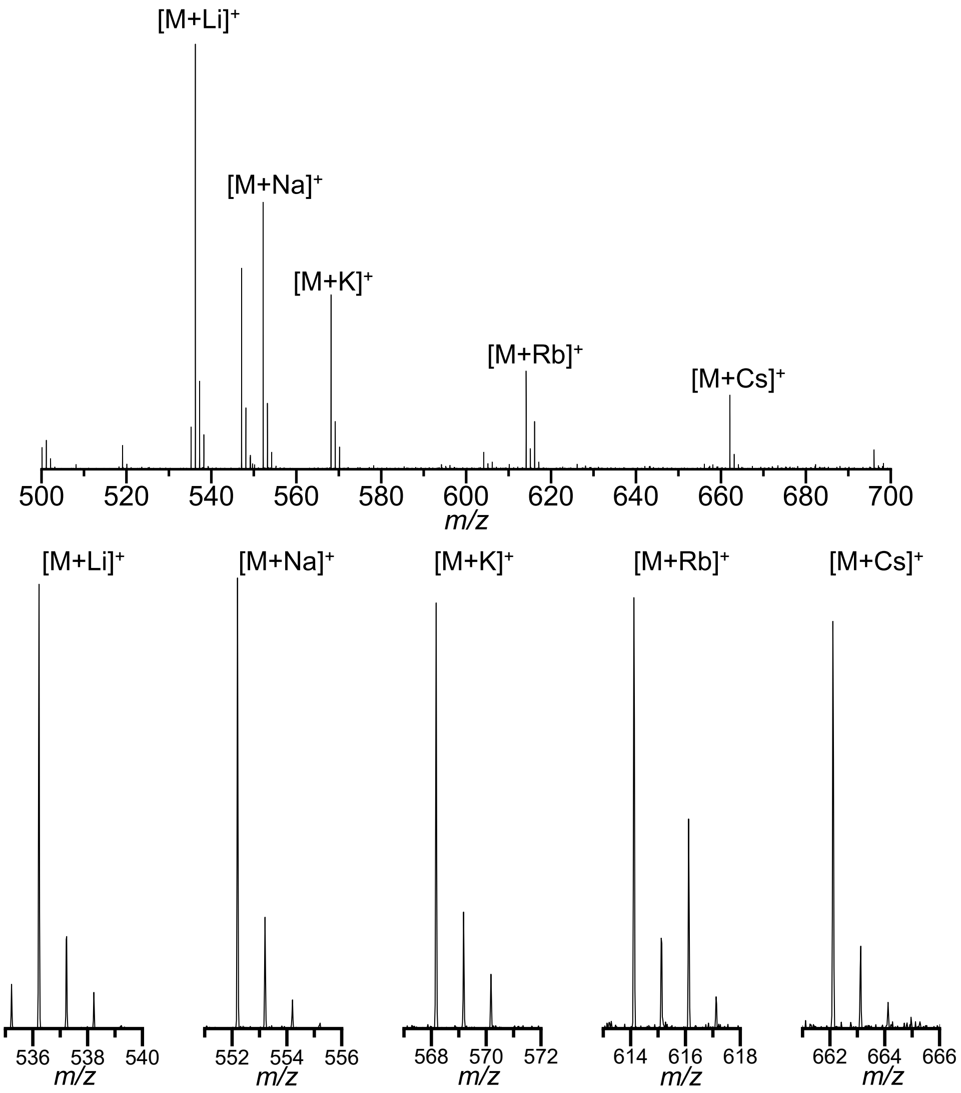

Figure S.2: Mass spectrum of isomeric trisaccharide mix; Lewis A, Lewis X, H-type 1, and H-type 2, run in positive ion mode producing observed cationic adduct species [M + Li]+ at *m/z* 536.2, [M + Na]+ at *m/z* 552.1, [M + K]+ at *m/z* 568.1, [M + Rb]+ at *m/z* 614.1 and [M + Cs]+ at *m/z* 662.1.

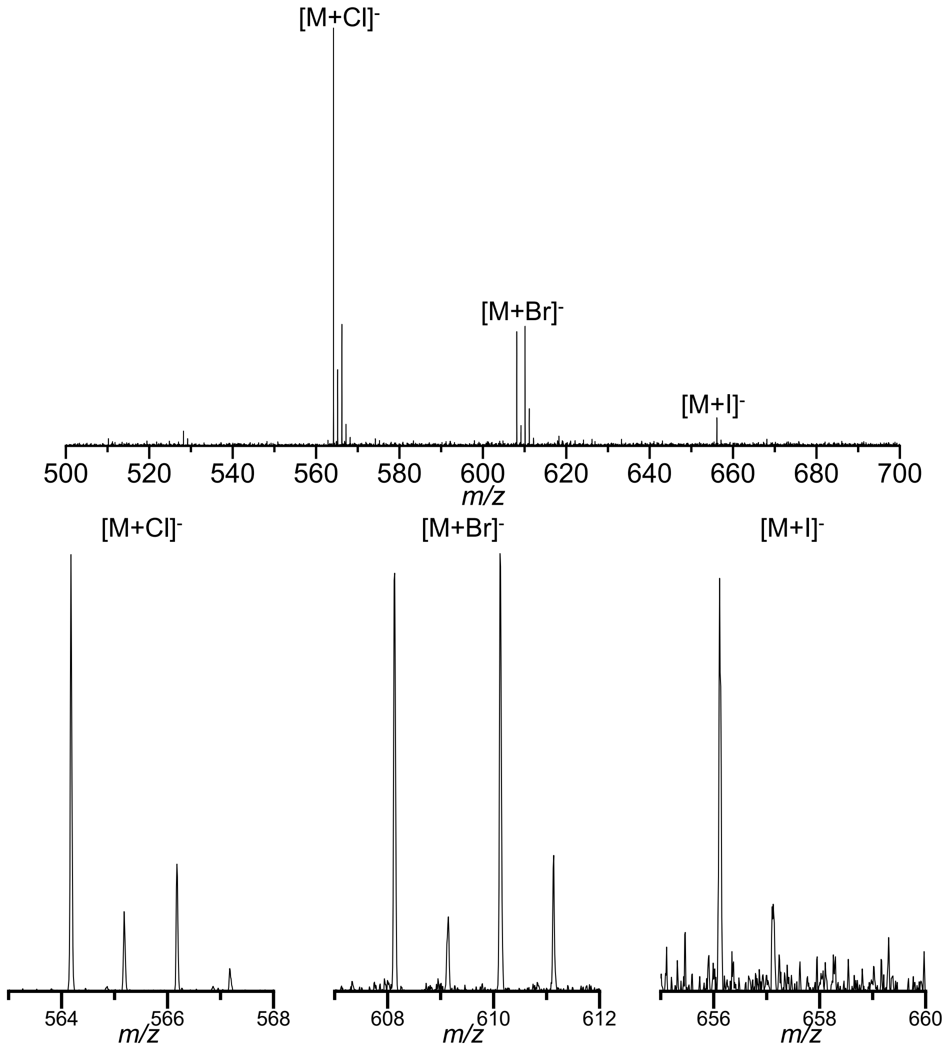

Figure S.3: Mass spectrum of isomeric trisaccharide mix; Lewis A, Lewis X, H-type 1, and H-type 2, run in negative ion mode producing observed anionic adduct species $[M + Cl]^-$ at *m/z* 564.1, $[M + Br]^-$ at *m/z* 608.1, and $[M + I]^-$ at *m/z* 656.0.

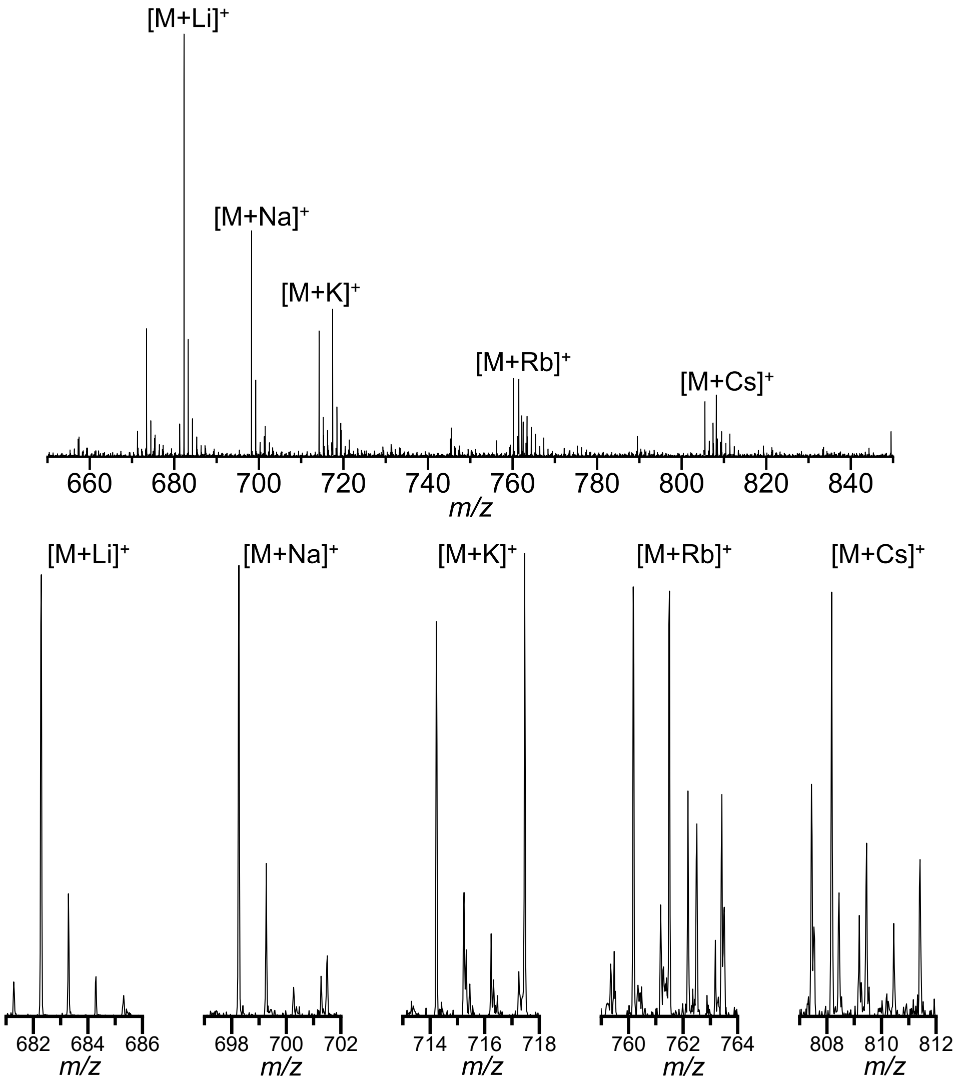

Figure S.4: Mass spectrum of isomeric tetrasaccharide mix; Lewis B and Lewis Y, run in positive ion mode producing observed cationic adduct species [M + Li]+ at *m/z* 682.2, [M + Na]+ at *m/z* 698.2, [M + K]+ at *m/z* 714.1, [M + Rb]+ at *m/z* 760.1 and [M + Cs]+ at *m/z* 808.1.

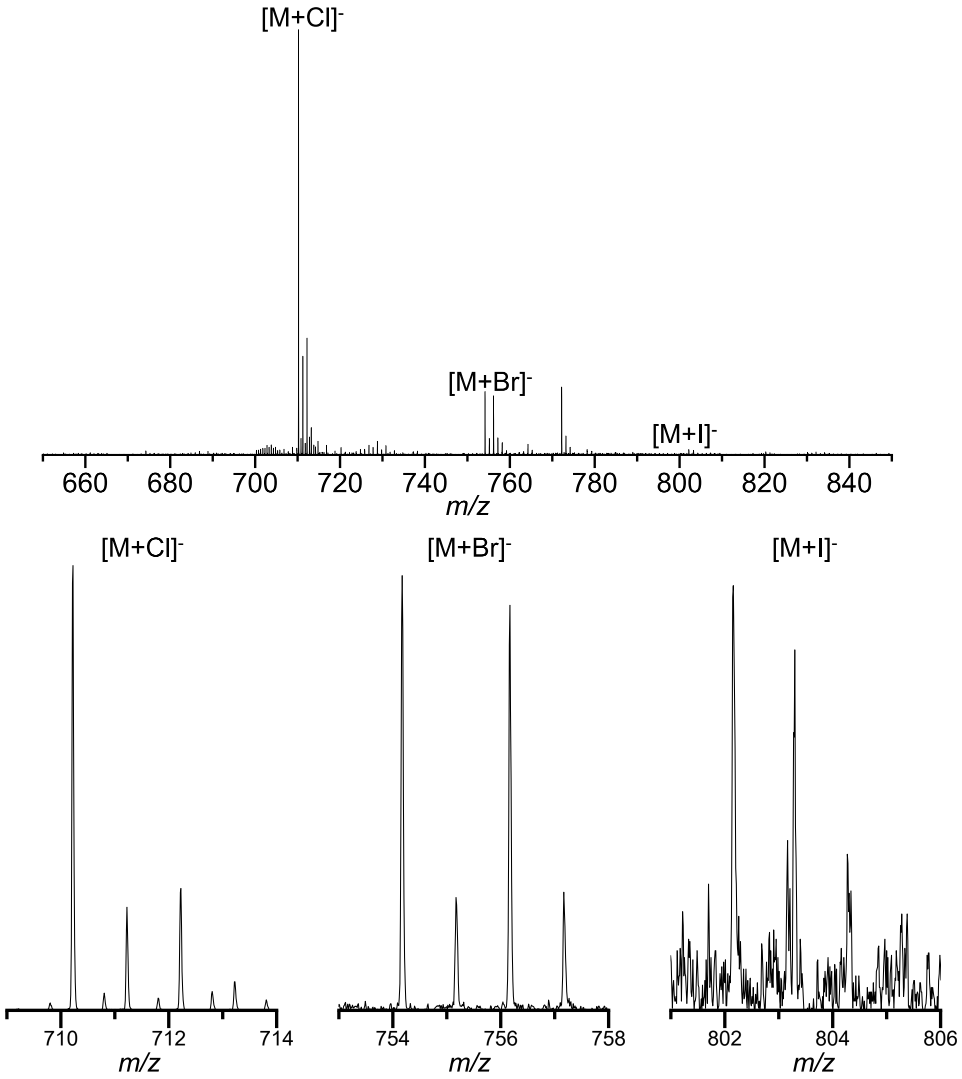

Figure S.5: Mass spectrum of isomeric tetrasaccharide mix; Lewis B and Lewis Y, run in negative ion mode producing observed anionic adduct species $[M + Cl]^-$ at *m/z* 710.2, $[M + Br]^-$ at *m/z* 754.1, and $[M + I]^-$ at *m/z* 802.1.

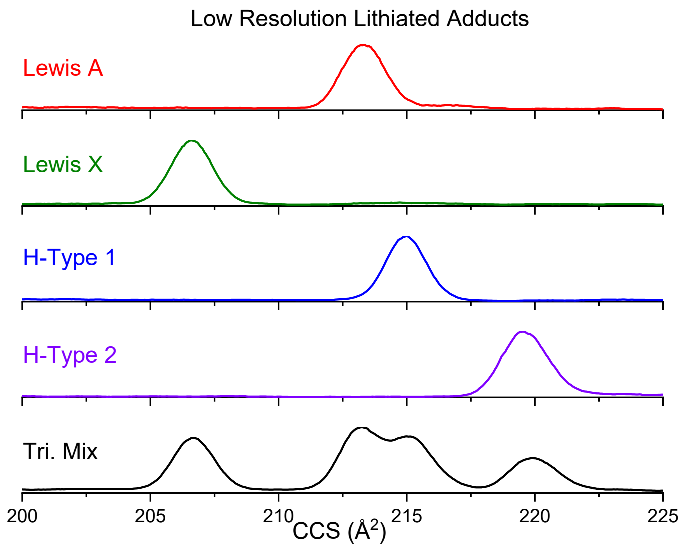

Figure S.6: Normalized low resolution (ΔV= 200V, S_R= 0.40 V/ms) ion mobility spectra for the trisaccharide $[M+Li]^+$ (*m/z* 536.2) cationic adduct species.

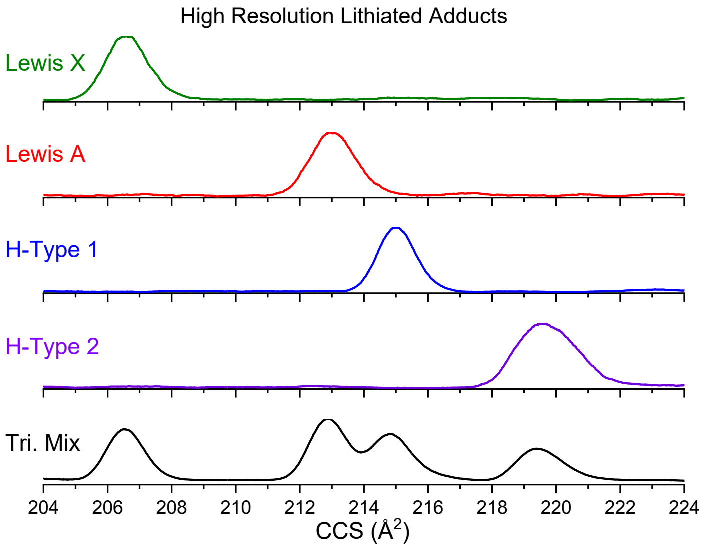

Figure S.7: Normalized high resolution (ΔV= 25V, S_R= 0.05 V/ms) ion mobility spectra for the trisaccharide $[M+Li]^+$ (*m/z* 536.2) cationic adduct species.

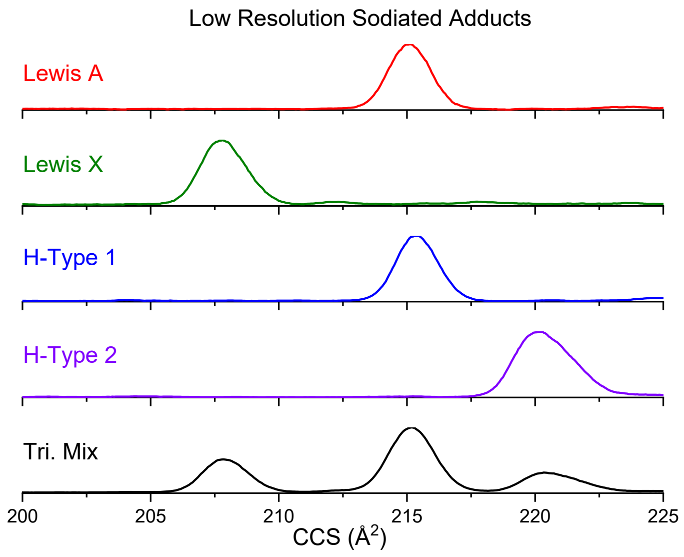

Figure S.8: Normalized low resolution (ΔV= 200V, S_R= 0.40 V/ms) ion mobility spectra for the trisaccharide $[M+Na]^+$ (*m/z* 552.1) cationic adduct species.

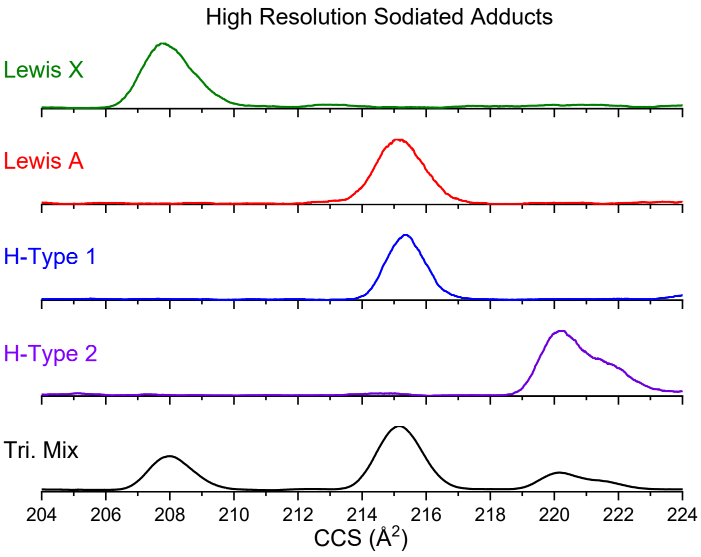

Figure S.9: Normalized high resolution (ΔV= 25V, S_R= 0.05 V/ms) ion mobility spectra for the trisaccharide $[M+Na]^+$ (*m/z* 552.1) cationic adduct species

.

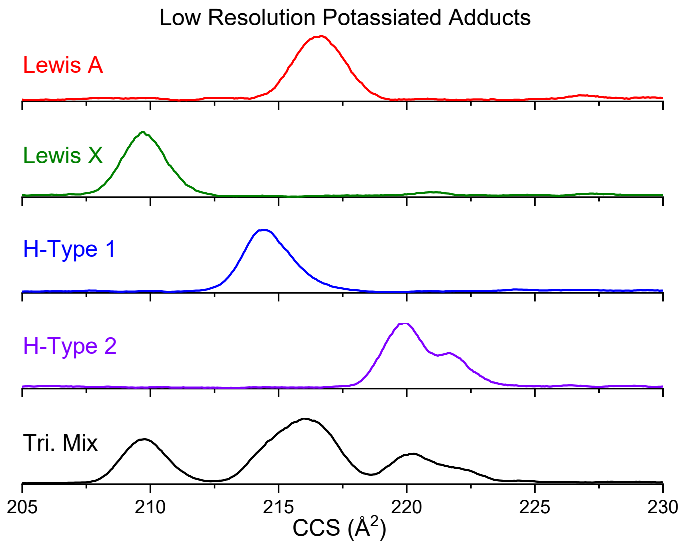

Figure S.10: Normalized low resolution (ΔV= 200V, S_R= 0.40 V/ms) ion mobility spectra for the trisaccharide $[M+K]^+$ (*m/z* 568.1) cationic adduct species.

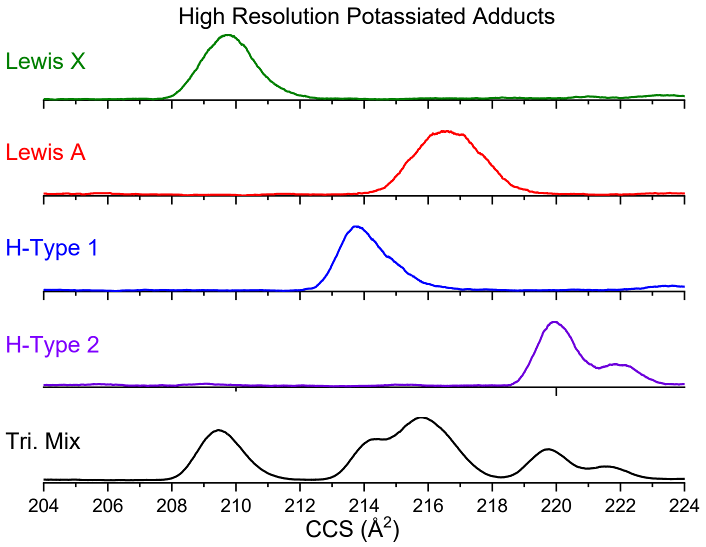

Figure S.11: Normalized high resolution (ΔV= 25V, S_R= 0.05 V/ms) ion mobility spectra for the trisaccharide $[M+K]^+$ (*m/z* 568.1) cationic adduct species.

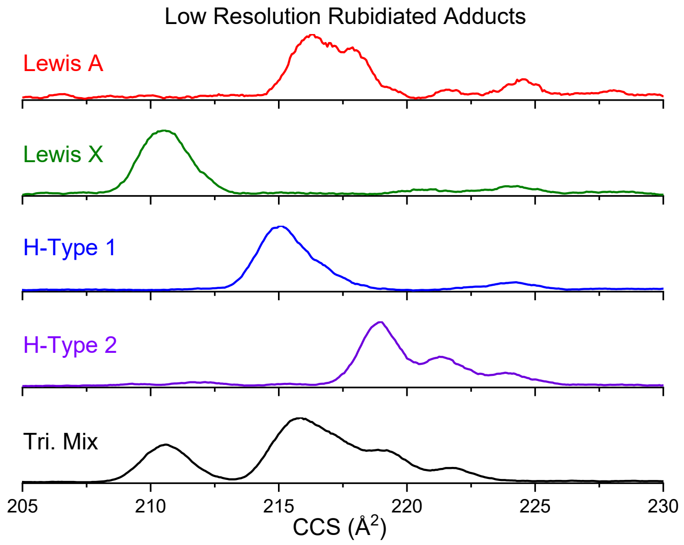

Figure S.12: Normalized low resolution (ΔV= 200V, S_R= 0.40 V/ms) ion mobility spectra for the trisaccharide $[M+Rb]^+$ (*m/z* 614.1) cationic adduct species.

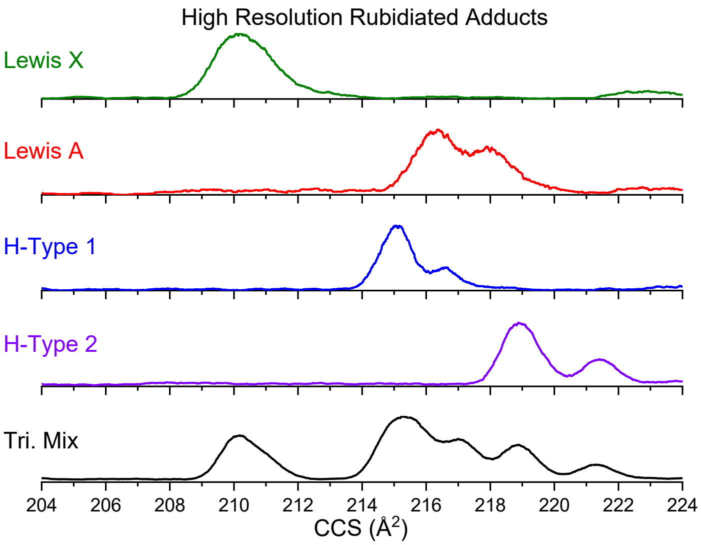

Figure S.13: Normalized high resolution (ΔV= 25V, S_R= 0.05 V/ms) ion mobility spectra for the trisaccharide $[M+Rb]^+$ (*m/z* 614.1) cationic adduct species.

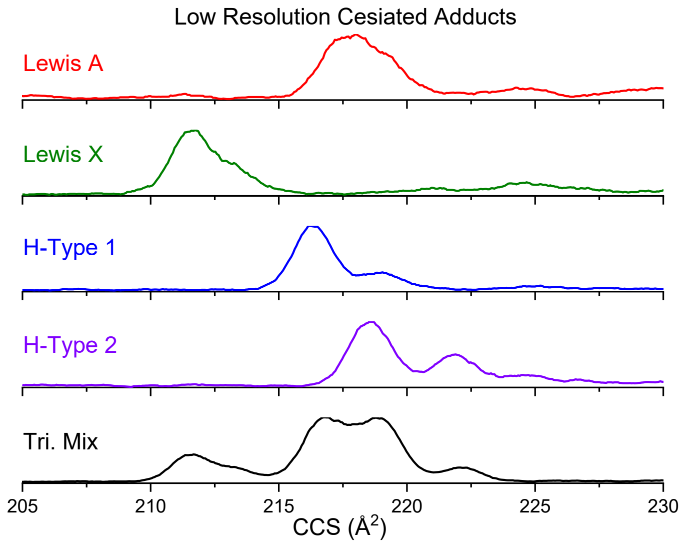

Figure S.14: Normalized low resolution (ΔV= 200V, S_R= 0.40 V/ms) ion mobility spectra for the trisaccharide $[M+Cs]^+$ (*m/z* 662.1) cationic adduct species.

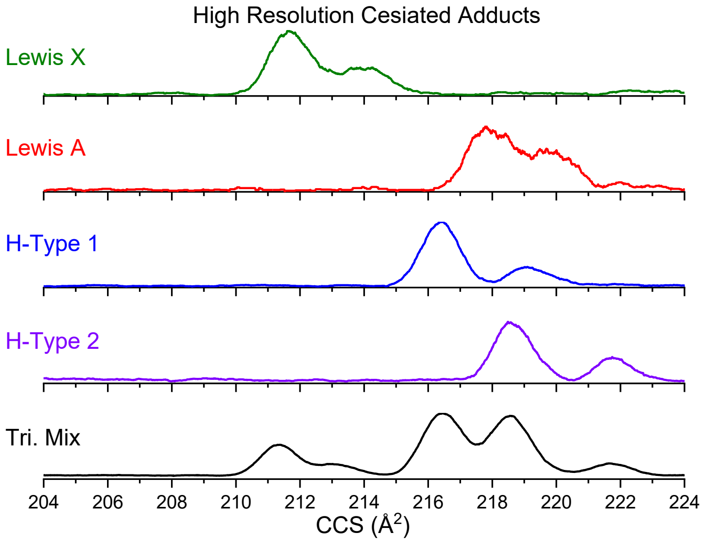

Figure S.15: Normalized high resolution (ΔV= 25V, S_R= 0.05 V/ms) ion mobility spectra for the trisaccharide $[M+Cs]^+$ (*m/z* 662.1) cationic adduct species.

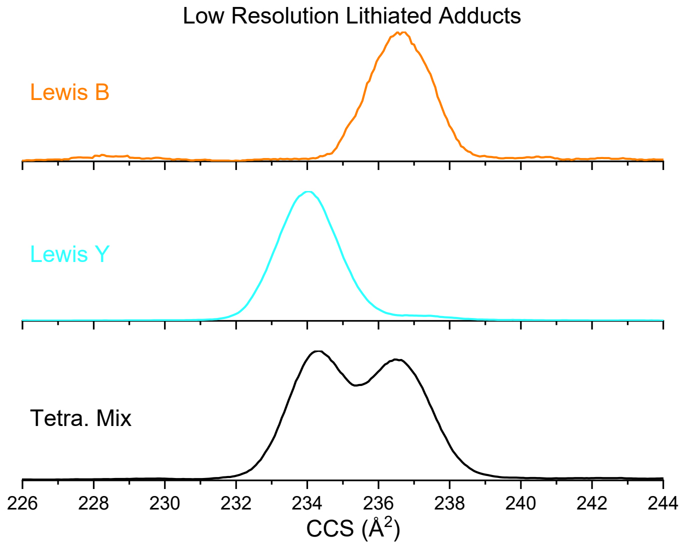

Figure S.16: Normalized low resolution (ΔV= 200V, S_R= 0.40 V/ms) ion mobility spectra for the tetrasaccharide $[M+Li]^+$ (*m/z* 682.2) cationic adduct species.

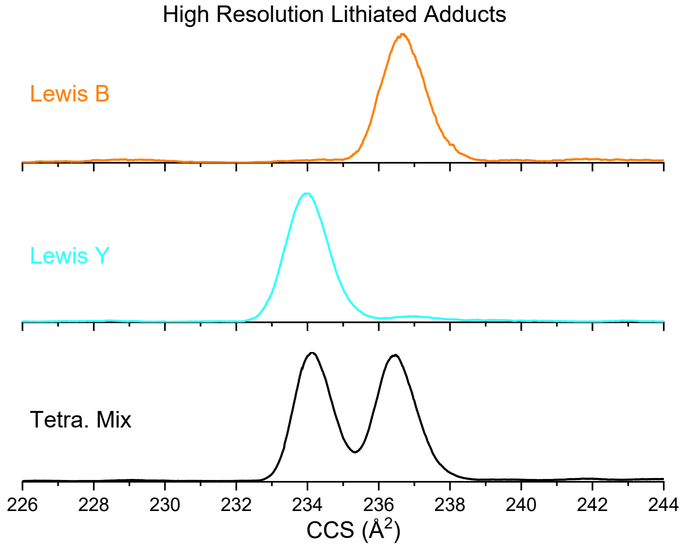

Figure S.17: Normalized high resolution (ΔV= 25V, S_R= 0.05 V/ms) ion mobility spectra for the tetrasaccharide $[M+Li]^+$ (*m/z* 682.2) cationic adduct species.

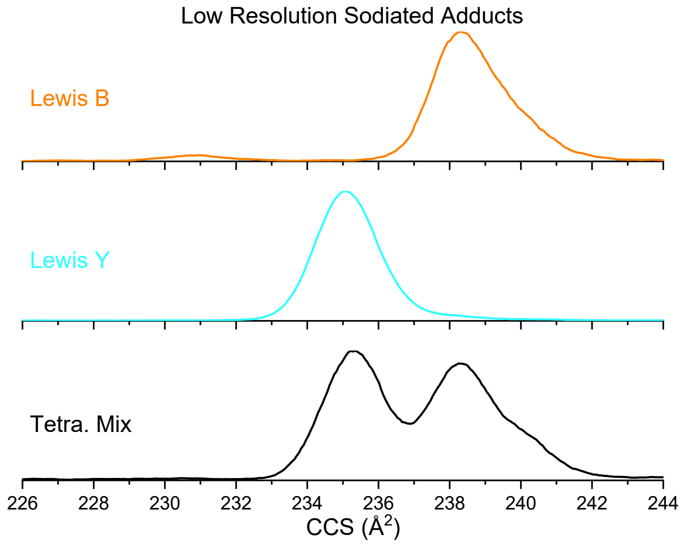

Figure S.18: Normalized low resolution (ΔV= 200V, S_R= 0.40 V/ms) ion mobility spectra for the tetrasaccharide $[M+Na]^+$ (*m/z* 698.2) cationic adduct species.

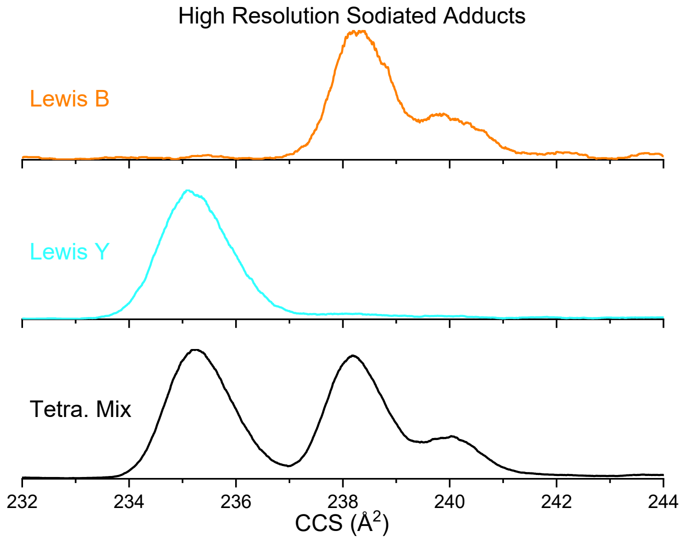

Figure S.19: Normalized high resolution (ΔV= 25V, S_R= 0.05 V/ms) ion mobility spectra for the tetrasaccharide $[M+Na]^+$ (*m/z* 698.2) cationic adduct species.

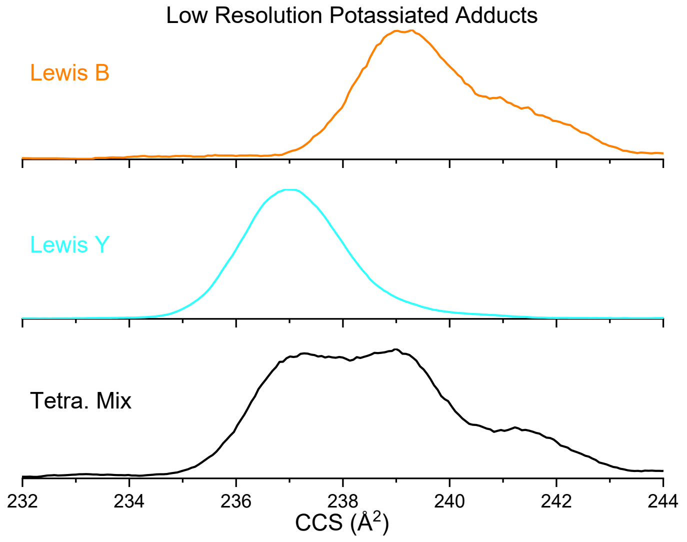

Figure S.20: Normalized low resolution (ΔV= 200V, S_R= 0.40 V/ms) ion mobility spectra for the tetrasaccharide $[M+K]^+$ (*m/z* 714.2) cationic adduct species.

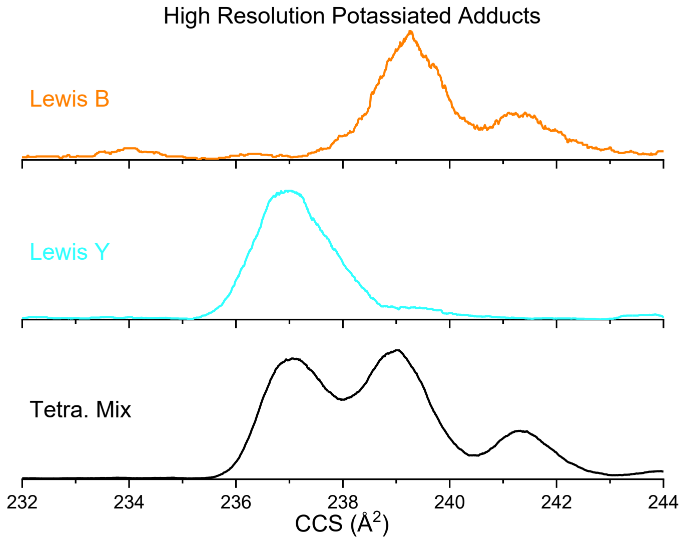

Figure S.21: Normalized high resolution (ΔV= 25V, S_R= 0.05 V/ms) ion mobility spectra for the tetrasaccharide $[M+K]^+$ (*m/z* 714.2) cationic adduct species.

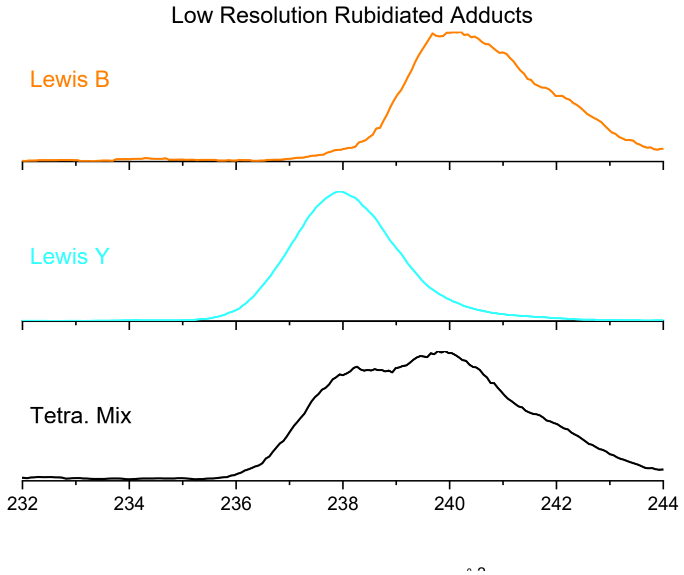

Figure S.22: Normalized low resolution (ΔV= 200V, S_R= 0.40 V/ms) ion mobility spectra for the tetrasaccharide $[M+Rb]^+$ (*m/z* 760.1) cationic adduct species.

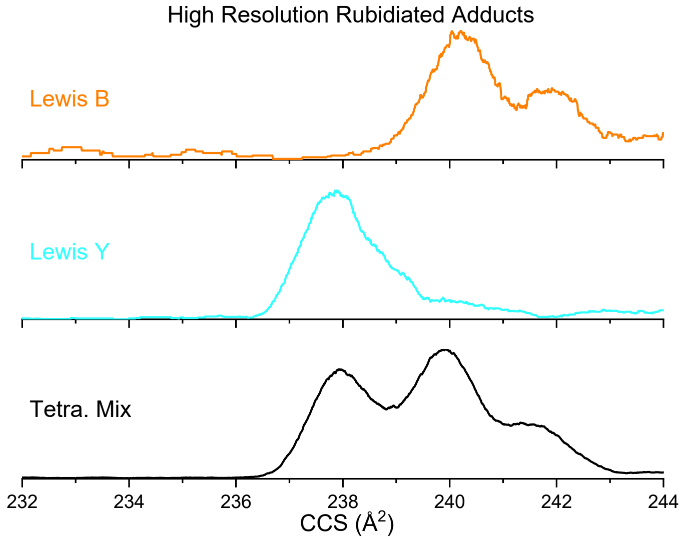

Figure S.23: Normalized high resolution (ΔV= 25V, S_R= 0.05 V/ms) ion mobility spectra for the tetrasaccharide $[M+Rb]^+$ (*m/z* 760.1) cationic adduct species.

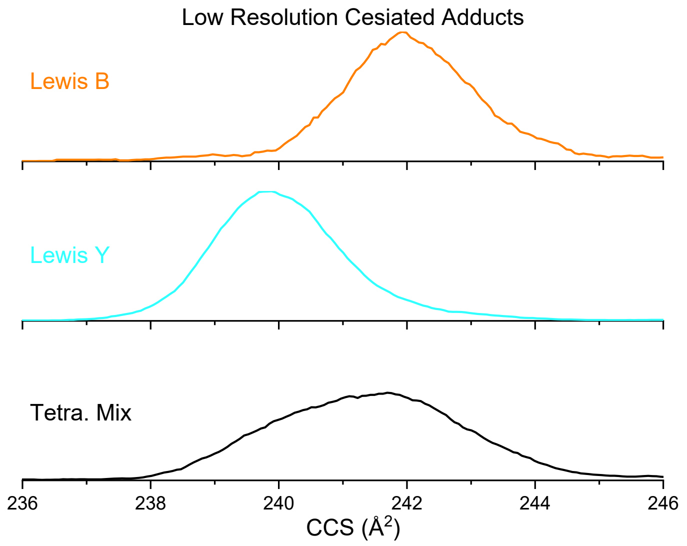

Figure S.24: Normalized low resolution (ΔV= 200V, S_R= 0.40 V/ms) ion mobility spectra for the tetrasaccharide $[M+Cs]^+$ (*m/z* 808.1) cationic adduct species.

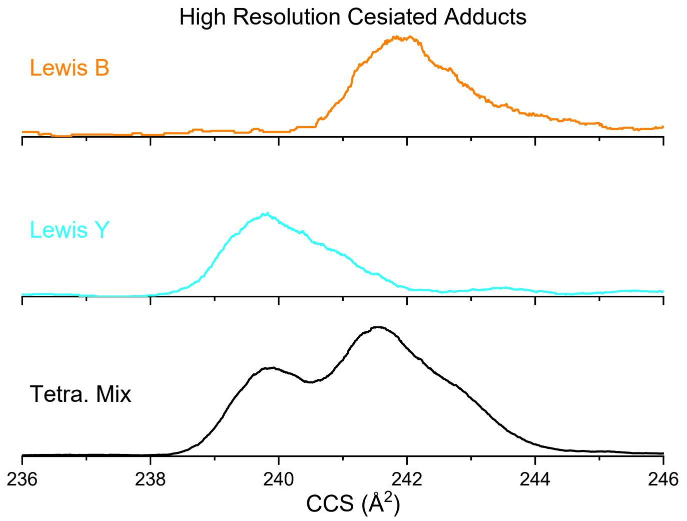

Figure S.25: Normalized high resolution (ΔV= 25V, S_R= 0.05 V/ms) ion mobility spectra for the tetrasaccharide $[M+Cs]^+$ (*m/z* 808.1) cationic adduct species.

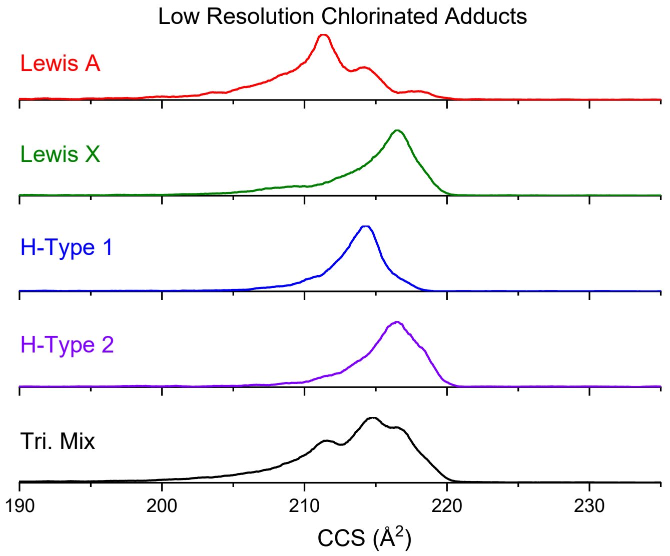

Figure S.26: Normalized low resolution (ΔV= 150V, S_R= 0.30 V/ms) ion mobility spectra for the trisaccharide $[M+Cl]^-$ (*m/z* 564.1) anionic adduct species.

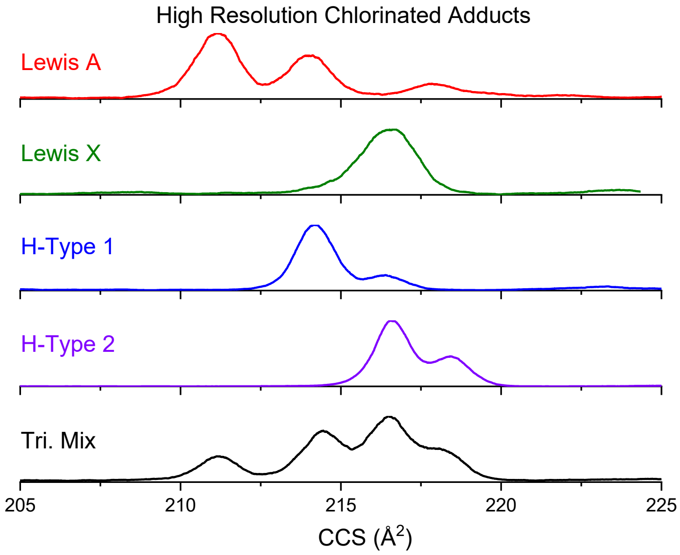

Figure S.27: Normalized high resolution (ΔV= 25V, S_R= 0.05 V/ms) ion mobility spectra for the trisaccharide $[M+Cl]^-$ (*m/z* 564.1) anionic adduct species.

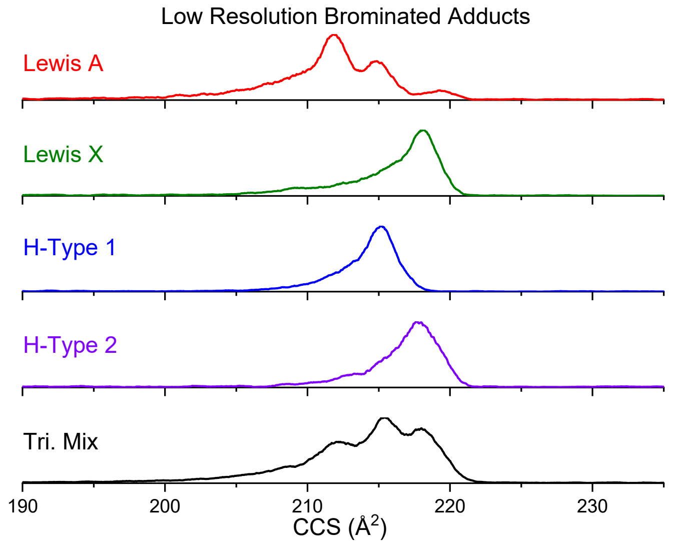

Figure S.28: Normalized low resolution (ΔV= 150V, S_R= 0.30 V/ms) ion mobility spectra for the trisaccharide $[M+Br]^-$ (*m/z* 610.1) anionic adduct species.

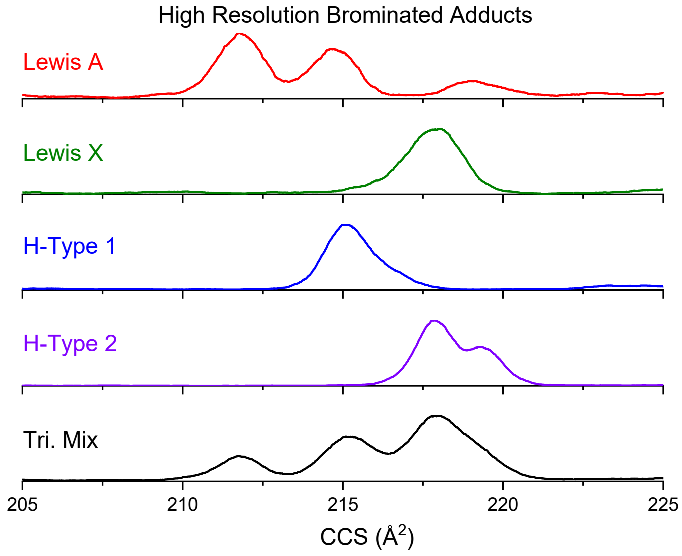

Figure S.29: Normalized high resolution (ΔV= 25V, S_R= 0.05 V/ms) ion mobility spectra for the trisaccharide $[M+Br]^-$ (*m/z* 610.1) anionic adduct species.

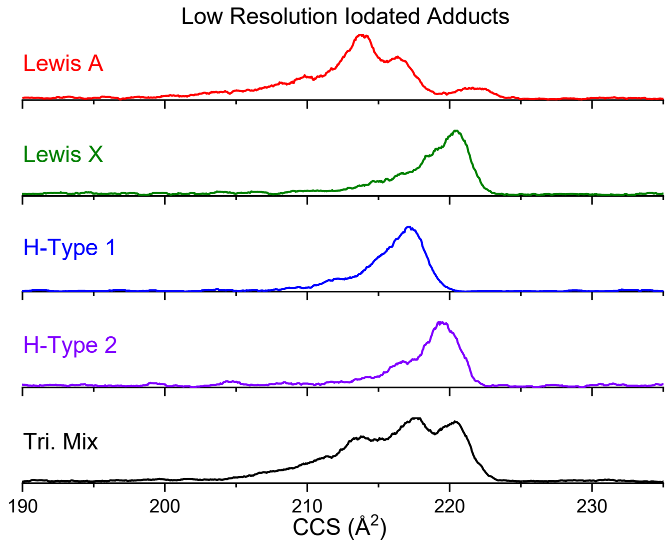

Figure S.30: Normalized low resolution (ΔV= 150V, S_R= 0.30 V/ms) ion mobility spectra for the trisaccharide $[M+I]^-$ (*m/z* 656.1) anionic adduct species.

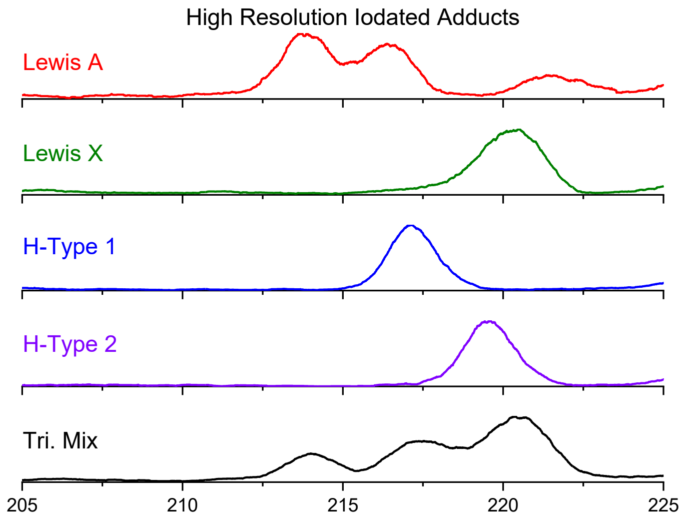

Figure S.31: Normalized high resolution (ΔV= 25V, S_R= 0.05 V/ms) ion mobility spectra for the trisaccharide $[M+I]^-$ (*m/z* 656.1) anionic adduct species.

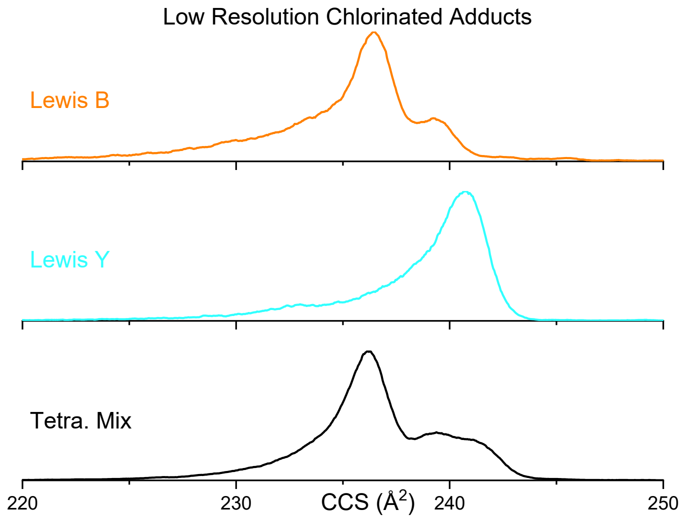

Figure S.32: Normalized low resolution (ΔV= 150V, S_R= 0.30 V/ms) ion mobility spectra for the tetrasaccharide $[M+Cl]^-$ (*m/z* 710.2) anionic adduct species.

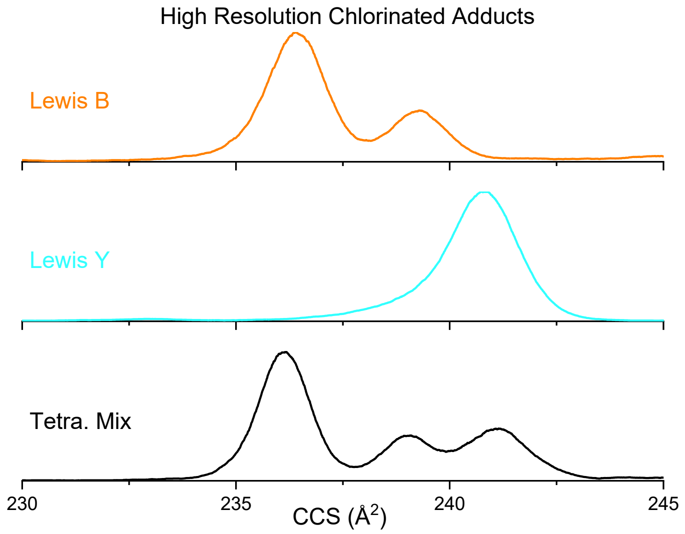

Figure S.33: Normalized high resolution (ΔV= 25V, S_R= 0.05 V/ms) ion mobility spectra for the tetrasaccharide $[M+Cl]^-$ (*m/z* 710.2) anionic adduct species.

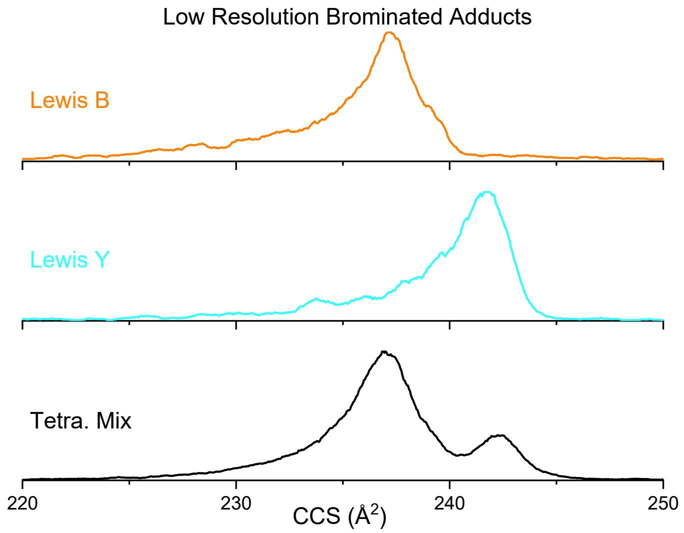

Figure S.34: Normalized low resolution (ΔV= 150V, S_R= 0.30 V/ms) ion mobility spectra for the tetrasaccharide $[M+Br]^-$ (*m/z* 756.1) anionic adduct species.

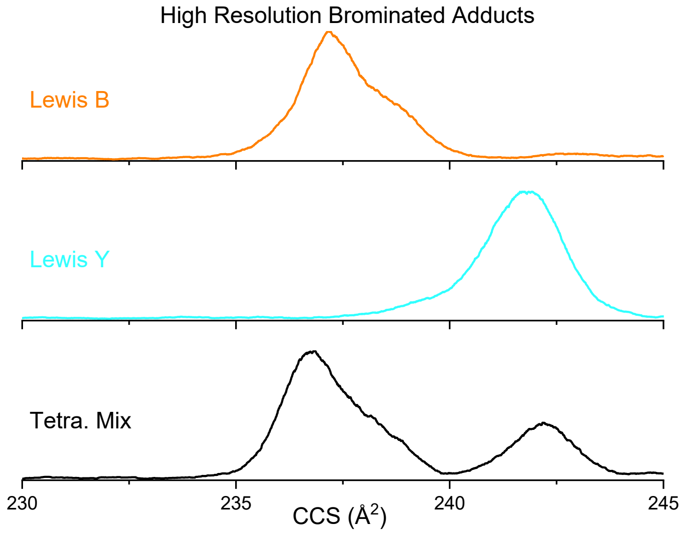

Figure S.35: Normalized high resolution (ΔV= 25V, S_R= 0.05 V/ms) ion mobility spectra for the tetrasaccharide $[M+Br]^-$ (*m/z* 756.1) anionic adduct species.

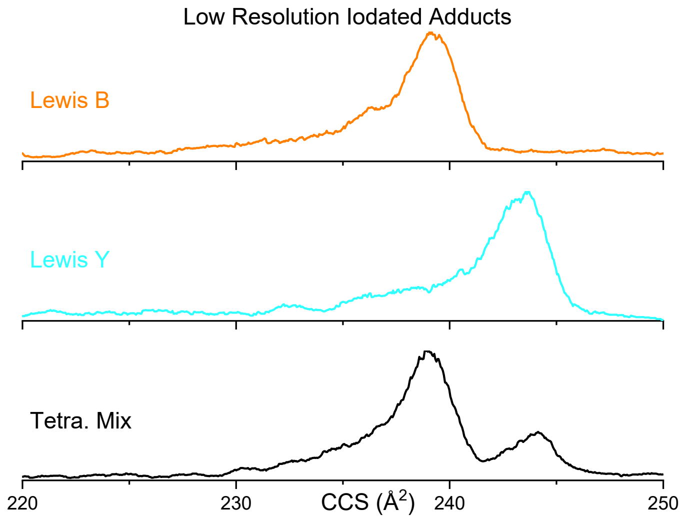

Figure S.36: Normalized low resolution (ΔV= 150V, S_R= 0.30 V/ms) ion mobility spectra for the tetrasaccharide $[M+I]^-$ (*m/z* 802.1) anionic adduct species.

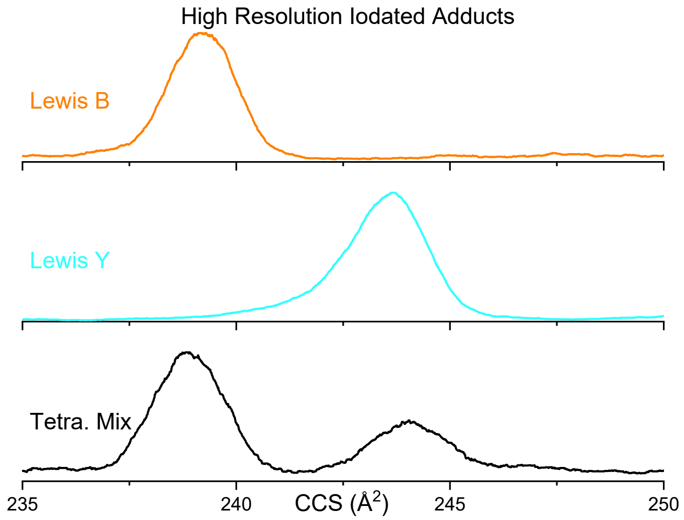

Figure S.37: Normalized high resolution (ΔV= 25V, S_R= 0.05 V/ms) ion mobility spectra for the tetrasaccharide $[M+I]^-$ (*m/z* 802.1) anionic adduct species.

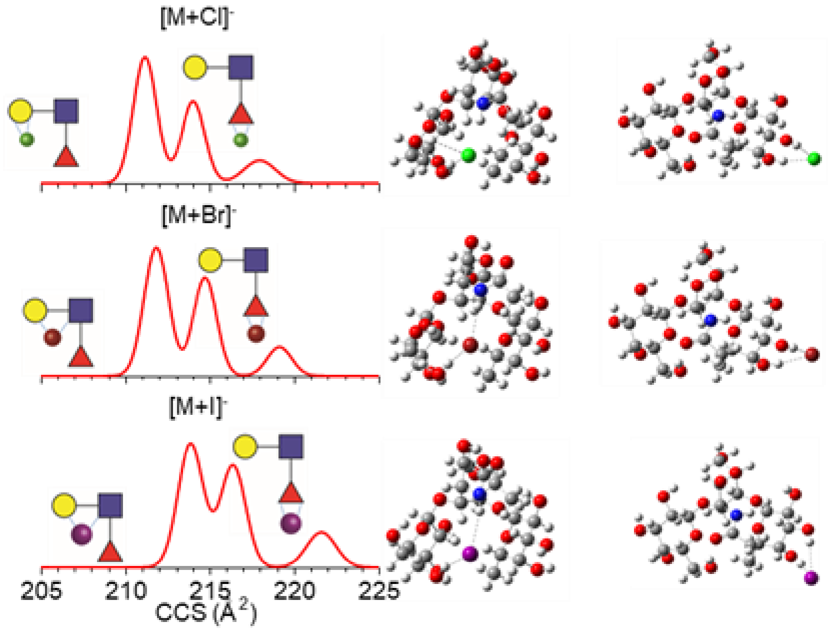

Figure S.38: Ion mobility spectra of oligosaccharide Lewis A, coordination motifs, and 3D theoretical structures of halogen anion species.

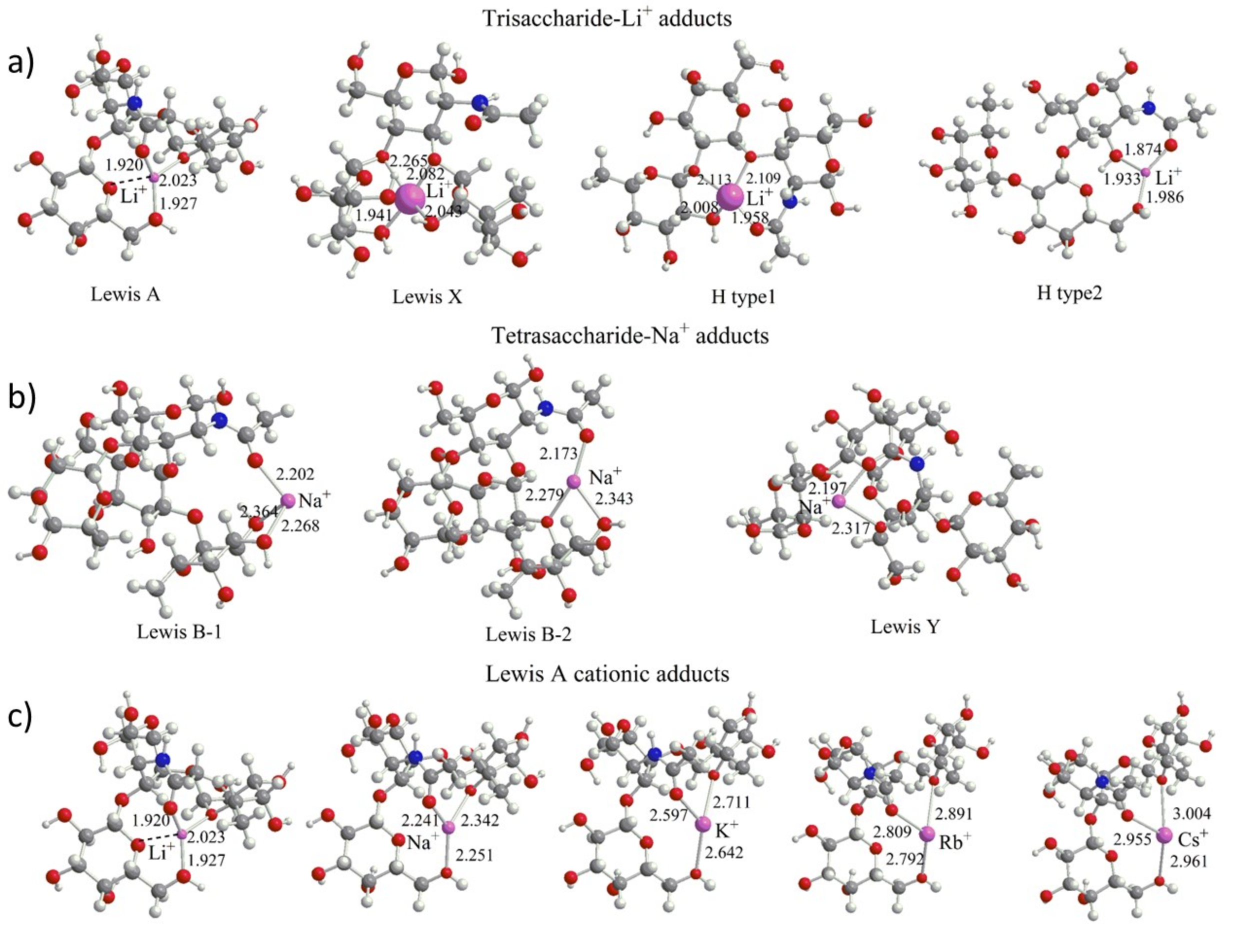

Figure S.39: 3D theoretical candidate structures with coordination site bond lengths for a) trisaccharide (Le^A, Le^X, BG-H^1, and BG-H^2) $[M+Li]^+$ adduct species, b) tetrasaccharide (Le^B and Le^Y) $[M+Na]^+$ adduct species, and c) Le^A bound with all cationic (Li^+, Na^+, K^+, Rb^+, and Cs^+) metal bound adducts.

Table S.1: Summary of experimental positive and negative mode $^{TIMS}CCS_{N2}$ and m/z values of IMS adducts compared to theoretical model CCS values (shown in parenthesis).

Oligosaccharide	Complex	Theoretical *m/z*	Experimental *m/z*	Experimental CCS (Å²)	Literature CCS (Å²)
Lewis A	$[M+Li]^+$	536.22	536.22	213.0 (222.3)	206.3[b]
	$[M+Na]^+$	552.19	552.19	215.2 (224.0)	232[a], 208.6[b]
	$[M+K]^+$	568.16	568.17	216.6 (225.2)	208.6[b]
	$[M+Rb]^+$	614.11	614.12	216.2, 217.9 (226.3, 226.9)	
	$[M+Cs]^+$	662.11	662.11	217.9, 219.8 (228.9, 230.6)	
	$[M+Cl]^-$	564.17	564.19	211.1, 214.0, 217.6 (210.9, 219.6)	
	$[M+Br]^-$	610.12	610.14	211.8, 214.7, 219.0 (211.8, 235.2)	
	$[M+I]^-$	656.10	656.13	213.8, 216.4, 221.5 (226.0, 251.3)	
Lewis X	$[M+Li]^+$	536.22	536.22	206.6 (216.6)	200.5[b]
	$[M+Na]^+$	552.19	552.19	207.9	215[a], 202.8[b]
	$[M+K]^+$	568.16	568.17	209.8	204.0[b]
	$[M+Rb]^+$	614.11	614.12	210.3	
	$[M+Cs]^+$	662.11	662.11	211.7, 213.9	
	$[M+Cl]^-$	564.17	564.20	216.5	
	$[M+Br]^-$	610.12	610.15	217.8	
	$[M+I]^-$	656.10	656.14	220.4	
H-Type 1	$[M+Li]^+$	536.22	536.22	215.0 (225.7)	209.8[b]
	$[M+Na]^+$	552.19	552.19	215.4	224[a], 210.9[b]
	$[M+K]^+$	568.16	568.17	214.0	208.6[b]
	$[M+Rb]^+$	614.11	614.12	215.1, 216.6	
	$[M+Cs]^+$	662.11	662.11	216.4, 219.1	
	$[M+Cl]^-$	564.17	564.20	214.2, 216.3	
	$[M+Br]^-$	610.12	610.15	215.1	
	$[M+I]^-$	656.10	656.14	217.2	
H-Type 2	$[M+Li]^+$	536.22	536.22	219.7 (223.4)	212.1[b]
	$[M+Na]^+$	552.19	552.19	220.1, 221.4	229[a], 212.1[b]
	$[M+K]^+$	568.16	568.17	220.0, 221.9	213.2[b]
	$[M+Rb]^+$	614.11	614.12	219.0, 221.4	
	$[M+Cs]^+$	662.11	662.11	218.6, 221.8	
	$[M+Cl]^-$	564.17	564.20	216.6, 218.4	
	$[M+Br]^-$	610.12	610.15	217.9, 219.3	
	$[M+I]^-$	656.10	656.14	219.6	
Lewis B	$[M+Li]^+$	682.27	682.28	236.7 (251.8)	231.7[b]
	$[M+Na]^+$	698.25	698.25	238.3, 240.0 (253.2, 254.1)	252[a], 232.9[b]

	$[M+K]^+$	714.22	714.23	239.2, 241.3 (256.1, 256.9)	232.9[b]
	$[M+Rb]^+$	760.17	760.17	240.2, 241.8 (257.0, 260.1)	
	$[M+Cs]^+$	808.16	808.17	241.9 (248.2)	
	$[M+Cl]^-$	710.23	710.23	236.4, 239.3 (243.9, 257.1)	
	$[M+Br]^-$	756.17	756.18	237.2, 238.5 (255.1, 266.1)	
	$[M+I]^-$	802.16	802.17	239.2 (265.0)	
Lewis Y	$[M+Li]^+$	682.27	682.28	234.0	229.4[b]
	$[M+Na]^+$	698.25	698.25	235.2 (244.3)	248[a], 230.6[b]
	$[M+K]^+$	714.22	714.23	237.0, 239.4	231.7[b]
	$[M+Rb]^+$	760.17	760.17	237.9	
	$[M+Cs]^+$	808.16	808.17	239.8	
	$[M+Cl]^-$	710.23	710.25	240.8	
	$[M+Br]^-$	756.17	756.20	241.8	
	$[M+I]^-$	802.16	802.18	243.6	

[a]Hofmann et al.[75], [b]Manz et al.[76](converted from $^{DT}CCS_{He}$)

Table S.2: Summary of experimental positive and negative mode $^{TIMS}CCS_{N2}$ and *m/z* values of IMS adducts with calculated resolving power (*R*) and resolution (*r*) values.

Oligosaccharide	Complex	*m/z*	CCS (Å^2)	Resolving Power (*R*)	Resolution (*r*)
Lewis X	$[M+Li]^+$	536.22	206.6	136.0	2.42 $(Le^A)_1$, 3.46 $(BG\text{-}H^1)_1$, 4.25 $(BG\text{-}H^2)_1$
	$[M+Na]^+$	552.19	207.9	118.2	2.47 $(Le^A)_1$, 2.82 $(BG\text{-}H^1)_1$, 4.81 $(BG\text{-}H^2)_1$
	$[M+K]^+$	568.17	209.8	113.5	1.93 $(Le^A)_1$, 1.39 $(BG\text{-}H^1)_1$, 3.84 $(BG\text{-}H^2)_1$
	$[M+Rb]^+$	614.12	210.3	103.5	2.05 $(Le^A)_1$, 1.78 $(BG\text{-}H^1)_1$, 3.07 $(BG\text{-}H^2)_1$
	$[M+Cs]^+$	662.11	211.7, 213.9	147.6, 115.1	2.52 $(Le^A)_1$, 2.03 $(BG\text{-}H^1)_1$, 2.96 $(BG\text{-}H^2)_1$
	$[M+Cl]^-$	564.20	216.5	105.1	0.41 $(Le^A)_3$, 0.04 $(BG\text{-}H^1)_2$, 0.05 $(BG\text{-}H^2)_1$
	$[M+Br]^-$	610.15	217.8	106.0	0.41 $(Le^A)_3$, 0.78 $(BG\text{-}H^1)_1$, 0.01 $(BG\text{-}H^2)_1$
	$[M+I]^-$	656.14	220.4	99.9	0.39 $(Le^A)_3$, 0.92 $(BG\text{-}H^1)_1$, 0.19 $(BG\text{-}H^2)_1$
Lewis A	$[M+Li]^+$	536.22	213.0	131.9	2.42 $(Le^X)_1$, 0.79 $(BG\text{-}H^1)_1$, 2.11 $(BG\text{-}H^2)_1$
	$[M+Na]^+$	552.19	215.2	126.6	2.47 $(Le^X)_1$, 0.07 $(BG\text{-}H^1)_1$, 1.98 $(BG\text{-}H^2)_1$
	$[M+K]^+$	568.17	216.6	92.0	1.93 $(Le^X)_1$, 0.78 $(BG\text{-}H^1)_1$, 1.09 $(BG\text{-}H^2)_1$
	$[M+Rb]^+$	614.12	216.2, 217.9	155.1, 130.4	2.05 $(Le^X)_1$, 0.17 $(BG\text{-}H^1)_2$, 1.89 $(BG\text{-}H^2)_1$
	$[M+Cs]^+$	662.11	217.9, 219.8	148.3, 125.5	1.14 $(Le^X)_2$, 0.54 $(BG\text{-}H^1)_2$, 0.32 $(BG\text{-}H^2)_1$
	$[M+Cl]^-$	564.19	211.1, 214.0, 217.6	129.0, 132.4, 99.0	1.70 $(Le^X)_1$, 1.18 $(BG\text{-}H^1)_1$, 2.17 $(BG\text{-}H^2)_1$
	$[M+Br]^-$	610.14	211.8, 214.7, 219.0	123.9, 132.9, 127.6	1.89 $(Le^X)_1$, 1.13 $(BG\text{-}H^1)_1$, 2.27 $(BG\text{-}H^2)_1$
	$[M+I]^-$	656.13	213.8, 216.4, 221.5	118.2, 122.4, 107.8	1.87 $(Le^X)_1$, 1.11 $(BG\text{-}H^1)_1$, 1.85 $(BG\text{-}H^2)_1$
H-Type 1	$[M+Li]^+$	536.22	215.0	159.4	0.79 $(Le^A)_1$, 3.46 $(Le^X)_1$, 1.59 $(BG\text{-}H^2)_1$
	$[M+Na]^+$	552.19	215.4	159.6	0.07 $(Le^A)_1$, 3.46 $(Le^X)_1$, 2.16 $(BG\text{-}H^2)_1$
	$[M+K]^+$	568.17	214.0	125.3	0.78 $(Le^A)_1$, 1.39 $(Le^X)_1$, 2.37 $(BG\text{-}H^2)_1$
	$[M+Rb]^+$	614.12	215.1, 216.6	189.2, 223.1	0.17 $(Le^A)_1$, 1.78 $(Le^X)_1$, 1.88 $(BG\text{-}H^2)_1$
	$[M+Cs]^+$	662.11	216.4, 219.1	165.9, 167.9	0.64 $(Le^A)_1$, 0.93 $(Le^X)_2$, 1.01 $(BG\text{-}H^2)_1$
	$[M+Cl]^-$	564.20	214.2, 216.3	151.4, 153.5	0.07 $(Le^A)_2$, 0.77 $(Le^X)_1$, 1.03 $(BG\text{-}H^2)_1$
	$[M+Br]^-$	610.15	215.1	115.1	0.17 $(Le^A)_2$, 0.78 $(Le^X)_1$, 1.03 $(BG\text{-}H^2)_1$
	$[M+I]^-$	656.14	217.2	127.3	0.26 $(Le^A)_2$, 0.92 $(Le^X)_1$, 0.80 $(BG\text{-}H^2)_1$
H-Type 2	$[M+Li]^+$	536.22	219.7	103.6	2.11 $(Le^A)_1$, 4.25 $(Le^X)_1$, 1.59 $(BG\text{-}H^1)_1$
	$[M+Na]^+$	552.19	220.1, 221.4	180.4, 105.7	1.98 $(Le^A)_1$, 4.81 $(Le^X)_1$, 2.16 $(BG\text{-}H^1)_1$
	$[M+K]^+$	568.17	220.0, 221.9	170.6, 144.7	1.09 $(Le^A)_1$, 3.84 $(Le^X)_1$, 2.37 $(BG\text{-}H^1)_1$
	$[M+Rb]^+$	614.12	219.0, 221.4	168.5, 190.8	0.39 $(Le^A)_2$, 3.07 $(Le^X)_2$, 1.14 $(BG\text{-}H^1)_2$
	$[M+Cs]^+$	662.11	218.6, 221.8	163.4, 203.6	0.32 $(Le^A)_1$, 1.75 $(Le^X)_1$, 0.22 $(BG\text{-}H^1)_2$
	$[M+Cl]^-$	564.20	216.6, 218.4	162.7, 157.0	0.16 $(Le^A)_3$, 0.05 $(Le^X)_1$, 0.11 $(BG\text{-}H^1)_2$
	$[M+Br]^-$	610.15	217.9, 219.3	151.4, 165.5	0.47 $(Le^A)_3$, 0.01 $(Le^X)_1$, 1.03 $(BG\text{-}H^1)_1$

	$[M+I]^-$	656.14	219.6	119.9	0.62 (Le^A)$_3$, 0.19 (Le^X)$_1$, 0.80 (BG-H^1)$_1$
Lewis B	$[M+Li]^+$	682.28	236.7	165.3	1.13 (Le^Y)$_1$
	$[M+Na]^+$	698.25	238.3, 240.0	211.3, 151.7	1.38 (Le^Y)$_1$
	$[M+K]^+$	714.23	239.2, 241.3	170.7, 163.3	0.08 (Le^Y)$_2$
	$[M+Rb]^+$	760.17	240.2, 241.8	184.7, 183.9	0.92 (Le^Y)$_1$
	$[M+Cs]^+$	808.17	241.9	139.4	0.69 (Le^Y)$_1$
	$[M+Cl]^-$	710.23	236.4, 239.3	141.2, 154.6	1.38 (Le^Y)$_1$
	$[M+Br]^-$	756.18	237.2, 238.5	136.4, 182.7	1.35 (Le^Y)$_1$
	$[M+I]^-$	802.17	239.2	135.2	1.31 (Le^Y)$_1$
Lewis Y	$[M+Li]^+$	682.28	234.0	170.0	1.13 (Le^B)$_1$
	$[M+Na]^+$	698.25	235.2	156.8	1.38 (Le^B)$_1$
	$[M+K]^+$	714.23	237.0, 239.4	150.2, 225.9	0.89 (Le^B)$_1$
	$[M+Rb]^+$	760.17	237.9	150.5	0.92 (Le^B)$_1$
	$[M+Cs]^+$	808.17	239.8	138.4	0.69 (Le^B)$_1$
	$[M+Cl]^-$	710.25	240.8	117.5	0.47 (Le^B)$_2$
	$[M+Br]^-$	756.20	241.8	110.3	0.96 (Le^B)$_2$
	$[M+I]^-$	802.18	243.6	110.1	1.31 (Le^B)$_2$

Note: Resolution values calculated using primary IMS band to nearest isomeric band as indicated by the subscript.

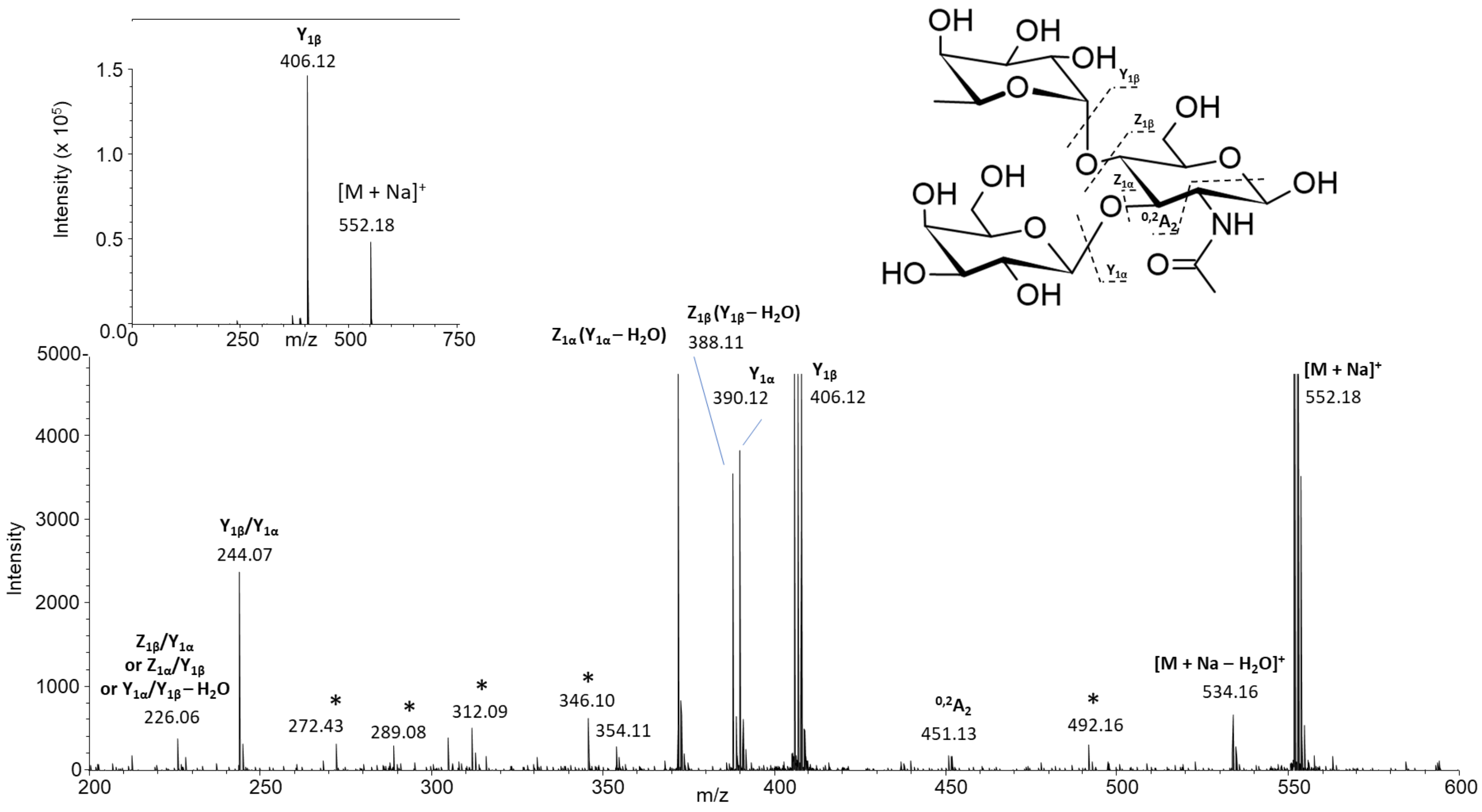

Figure S.40: MS2 (CID) Spectrum - Lewis A Trisaccharide $[M + Na]^+$

Table S.3: Observed Fragments in MS2 (CID) Spectrum - Lewis A Trisaccharide $[M + Na]^+$

Fragment	m/z	Ions
$Y_{1\alpha}Y_{1\beta}$ - H_2O	226.07	Na
$Z_{1\beta}Y_{1\alpha}$	226.07	Na
$Z_{1\alpha}Y_{1\beta}$	226.07	Na
$Y_{1\alpha}Y_{1\beta}$	244.08	Na
$^{1,5}X_{2\alpha}Y_{1\beta}$	272.08	Na
$^{1,5}X_{Fuc}Y_{1\alpha}$	272.07	Na
$^{2,5}AY_{1\beta}$	289.09	Na
$^{0,2}A_{GlcNAc}Y_{1\alpha}$	289.09	Na
$^{1,3}X_{GlcNAc}$	289.09	Na
$^{0,4}X_{GlcNAc}Z_{1\alpha}$	312.11	Na
$Z_{1\alpha}{}^{1,3}X_{Fuc}$	312.11	Na
$Z_{1\alpha}{}^{2,4}X_{Fuc}$	312.11	Na
$^{0,4}X_{GlcNAc}Y_{1\beta}$	346.11	Na
$^{0,4}X_{Gal}Y_{1\beta}$	346.11	Na
$^{1,3}X_{Gal}Y_{1\beta}$	346.11	Na
$^{2,4}X_{Gal}Y_{1\beta}$	346.11	Na
$Y_{1\alpha}{}^{0,4}X_{Fuc}$	346.11	Na
$Z_{1\alpha}$	372.13	Na
$Z_{1\beta}$	388.12	Na
$Y_{1\alpha}$	390.14	Na
$Y_{1\beta}$	406.13	Na
$^{0,2}A_{GlcNAc}$	451.14	Na
$^{0,4}X_{GlcNAc}$	492.17	Na
$^{1,3}X_{Fuc}$	492.17	Na
$^{2,4}X_{Fuc}$	492.17	Na
$^{0,4}X_{Gal}$	492.17	Na
$^{1,3}X_{Gal}$	492.17	Na
$^{2,4}X_{Gal}$	492.17	Na
$[M + Na - H_2O]^+$	534.17	Na
$[M + Na]^+$	552.19	Na

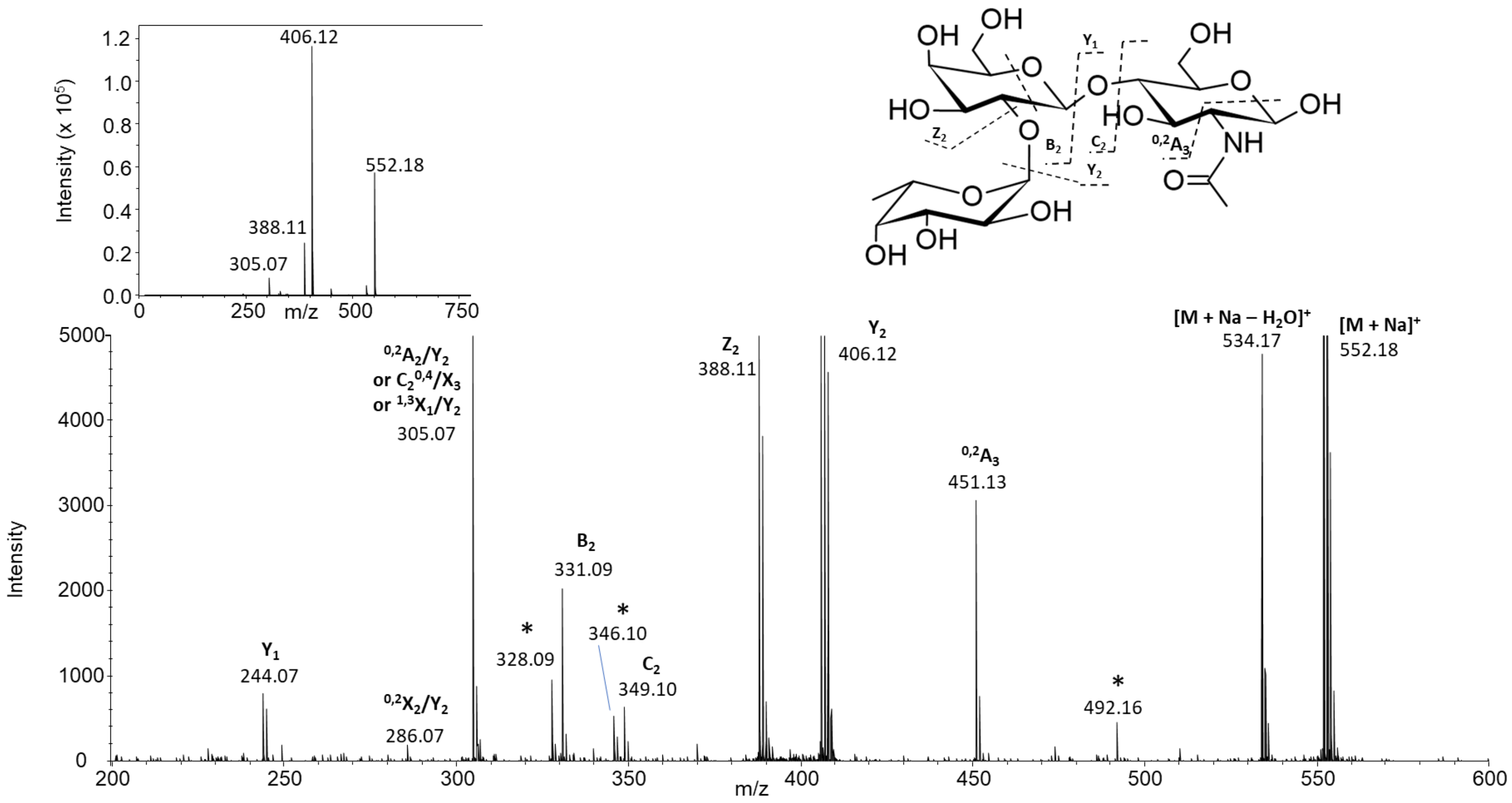

Figure S.41: MS2 (CID) Spectrum - H-type 2 Band 1 $[M + Na]^+$

Table S.4: Observed Fragments in MS2 (CID) Spectrum H Antigen Type 2 Trisaccharide $[M + Na]^+$ Band 1

Type	m/z	Ions
Y_1	244.08	Na
$^{0,2}X_2Y_2$	286.09	Na
$^{0,2}A_2Y_2$	305.08	Na
$C_2{}^{0,4}X_3$	305.08	Na
$^{1,3}X_1Y_2$	305.08	Na
$^{0,4}X_1Z_2$	328.10	Na
$^{0,4}X_2Z_2$	328.10	Na
$^{2,4}X_2Z_2$	328.10	Na
B_2	331.10	Na
$^{0,4}X_1Y_2$	346.11	Na
$^{0,4}X_2$	346.11	Na
$^{1,3}X_2$	346.11	Na
$^{2,4}X_2$	346.11	Na
C_2	349.11	Na
Z_2	388.12	Na
Y_2	406.13	Na
$^{0,2}A_3$	451.14	Na
$^{0,4}X_{GlcNAc}$	492.17	Na
$^{1,3}X_{Fuc}$	492.17	Na
$^{2,4}X_{Fuc}$	492.17	Na
$^{0,4}X_{Gal}$	492.17	Na
$^{2,4}X_{Gal}$	492.17	Na
$[M + Na - H_2O]^+$	534.17	Na
$[M + Na]^+$	552.19	Na

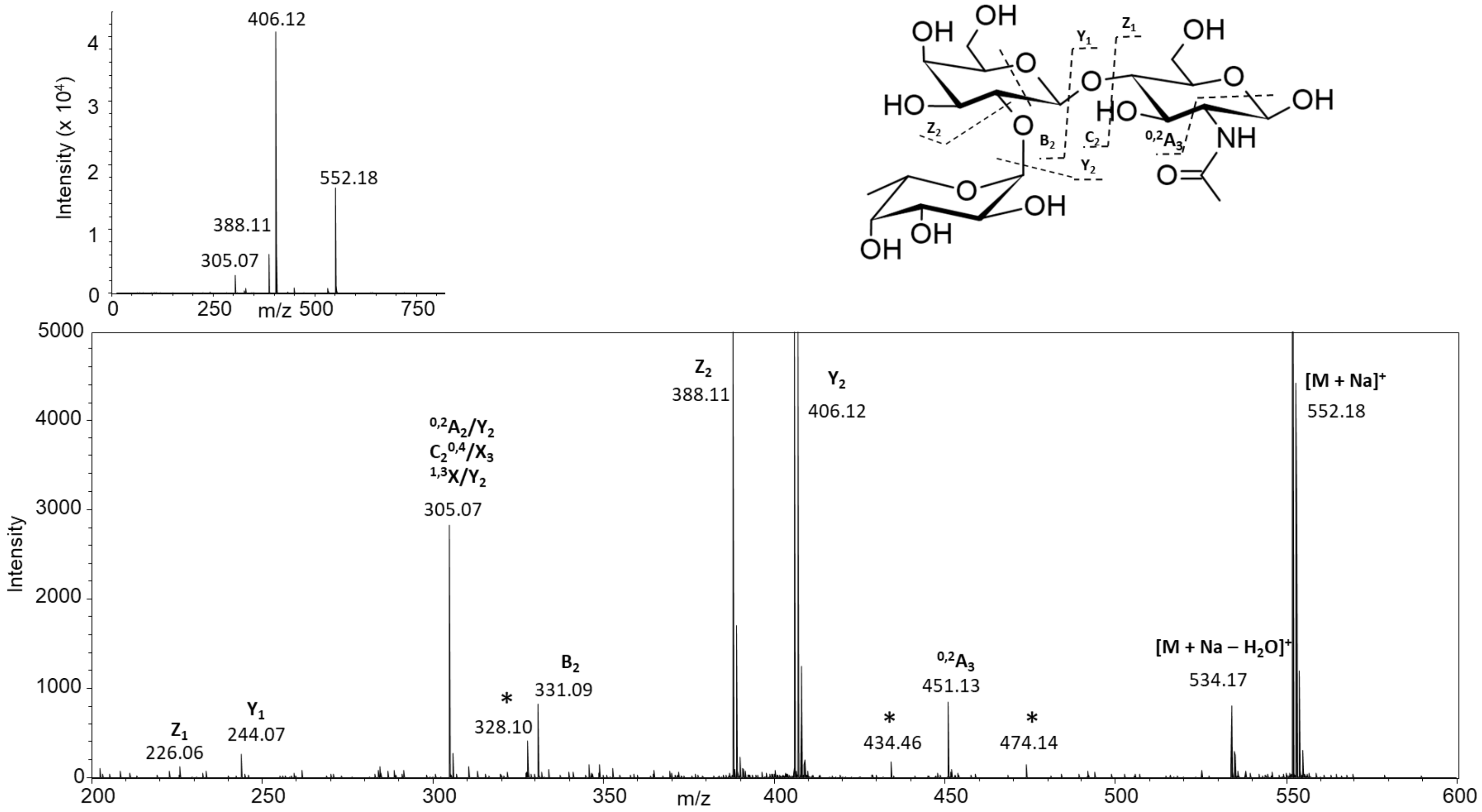

Figure S.42: MS2 (CID) Spectrum - H-type 2 Band 2 $[M + Na]^+$

Table S.5: Observed Fragments in MS2 (CID) Spectrum H Antigen Type 2 Trisaccharide [M + Na]+ Band 2

Type	m/z	Ions
Y_1	244.08	Na
$^{0,2}X_2Y_2$	286.09	Na
$^{0,2}A_2Y_2$	305.08	Na
$C_2{}^{0,4}X_3$	305.08	Na
$^{1,3}X_1Y_2$	305.08	Na
$^{0,4}X_1Z_2$	328.10	Na
$^{0,4}X_2Z_2$	328.10	Na
$^{2,4}X_2Z_2$	328.10	Na
B_2	331.10	Na
$^{0,4}X_1Y_2$	346.11	Na
$^{0,4}X_2$	346.11	Na
$^{1,3}X_2$	346.11	Na
$^{2,4}X_2$	346.11	Na
C_2	349.11	Na
Z_2	388.12	Na
Y_2	406.13	Na
$^{1,5}X_3$	434.13	Na
$^{0,2}A_3$	451.14	Na
$^{0,4}X_{GlcNAc}$	492.17	Na
$^{1,3}X_{Fuc}$	492.17	Na
$^{2,4}X_{Fuc}$	492.17	Na
$^{0,4}X_{Gal}$	492.17	Na
$^{2,4}X_{Gal}$	492.17	Na
$[M + Na - H_2O]^+$	534.17	Na
$[M + Na]^+$	552.19	Na

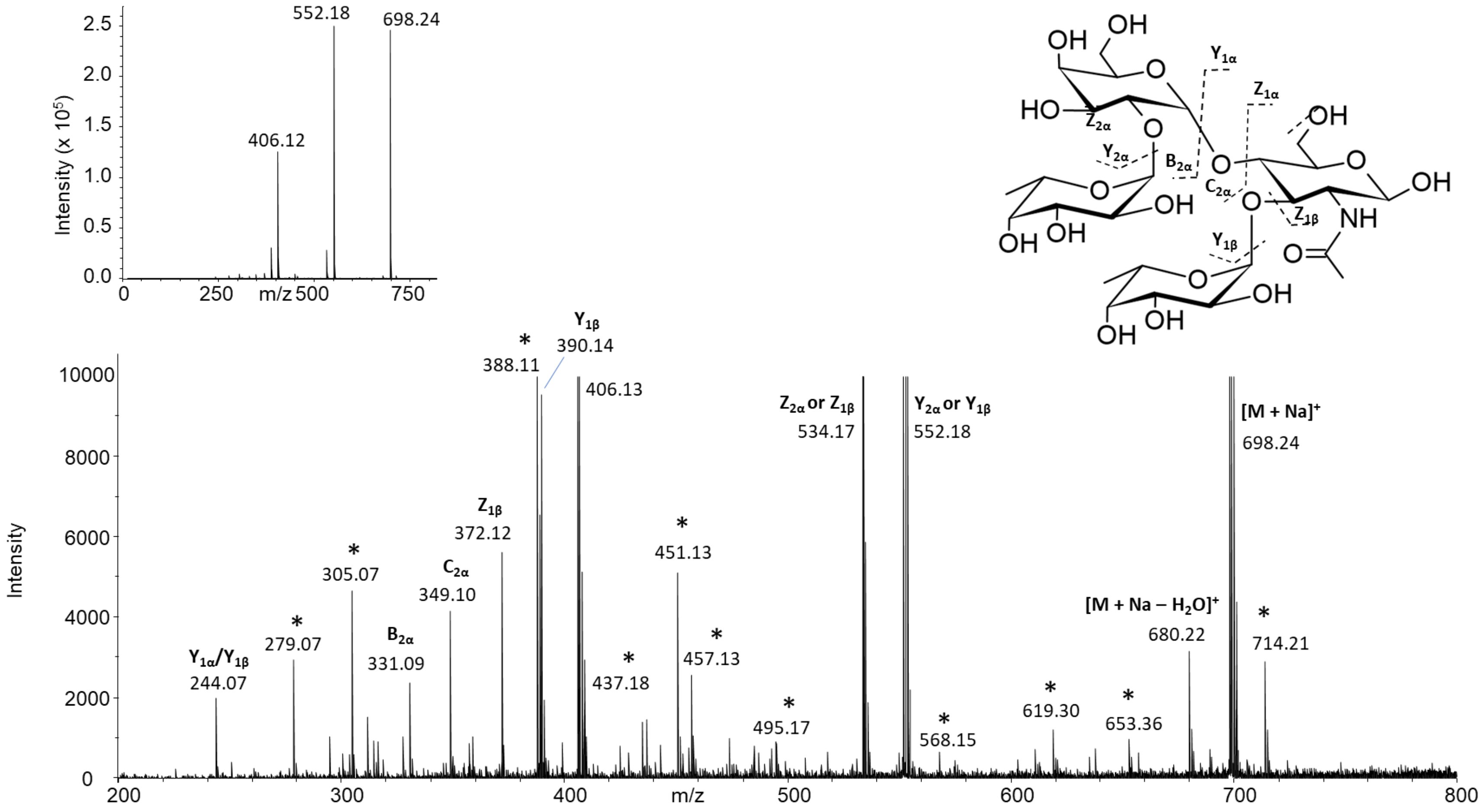

Figure S.43: MS2 (CID) Spectrum - Lewis Y Tetrasaccharide [M + Na]+

Table S.6: Observed Fragments in MS2 (CID) Spectrum Lewis Y Tetrasaccharide [M + Na]+

Type	m/z	Ions
$Y_{1\alpha}Y_{1\beta}$	244.08	Na
$C_{2\alpha}{}^{0,4}X_{Fuc}$	305.08	Na
$^{1,3}X_{GlcNAc}Y_{2\alpha}$	305.08	Na
$B_{2\alpha}$	331.10	Na
$C_{2\alpha}$	349.11	Na
$Z_{1\alpha}$	372.13	Na
$Y_{1\beta}Z_{2\alpha}$	388.12	Na
$Z_{1\beta}Y_{2\alpha}$	388.12	Na
$Y_{1\alpha}$	390.14	Na
$Y_{1\beta}Y_{2\alpha}$	406.13	Na
$^{0,2}A_{GlcNAc}Y_{2\alpha}$	451.14	Na
$^{0,2}A_{GlcNAc}Y_{1\beta}$	451.14	Na
$^{1,3}X_{GlcNAc}$	451.14	Na
$Z_{1\beta}$	534.18	Na
$Z_{2\alpha}$	534.18	Na
$Y_{1\beta}$	552.19	Na
$Y_{2\alpha}$	552.19	Na
[M+Na-H_2O]+	680.24	Na
[M+Na]+	698.25	Na

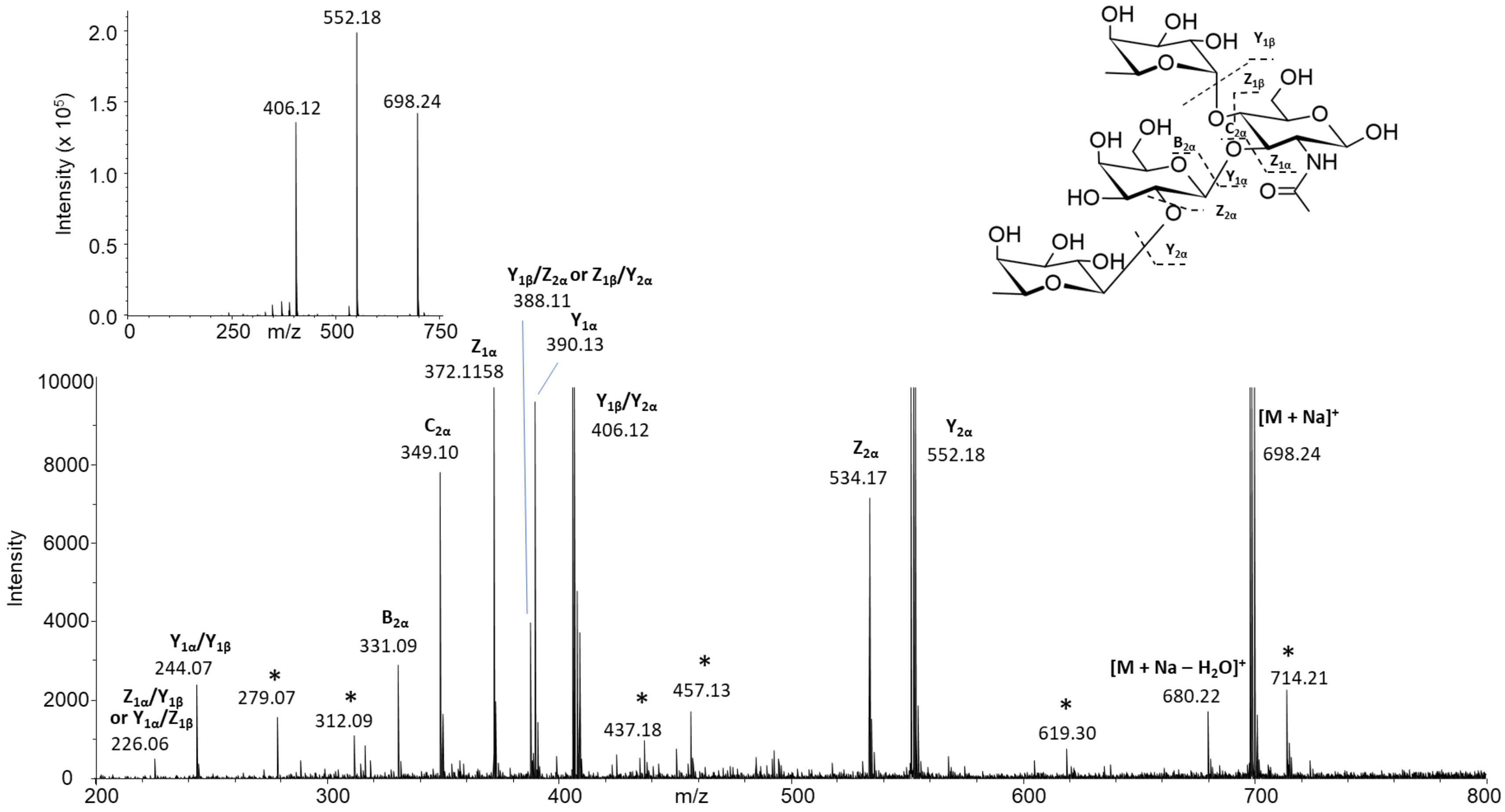

Figure S.44: MS2 (CID) Spectrum - Lewis B [M + Na]+ IMS 1

Table S.7: Observed Fragments in MS2 (CID) Spectrum Lewis B Tetrasaccharide [M + Na]+ IMS 1

Type	m/z	Ions
$Y_{1\alpha}Z_{1\beta}$	226.07	Na
$Z_{1\alpha}Y_{1\beta}$	226.07	Na
$Y_{1\alpha}Y_{1\beta}$	244.08	Na
$^{0,4}X_{GlcNAc}Z_{1\alpha}$	312.11	Na
$Z_{1\alpha}{}^{1,3}X_{Fuc}$	312.11	Na
$Z_{1\alpha}{}^{2,4}X_{Fuc}$	312.11	Na
$B_{2\alpha}$	331.10	Na
$C_{2\alpha}$	349.11	Na
$Z_{1\alpha}$	372.13	Na
$Y_{1\beta}Z_{2\alpha}$	388.12	Na
$Z_{1\beta}Y_{2\alpha}$	388.12	Na
$Y_{1\alpha}$	390.14	Na
$Y_{1\beta}Y_{2\alpha}$	406.13	Na
$Z_{2\alpha}$	534.18	Na
$Y_{2\alpha}$	552.19	Na
$[M+Na-H_2O]^+$	680.24	Na
$[M+Na]^+$	698.25	Na

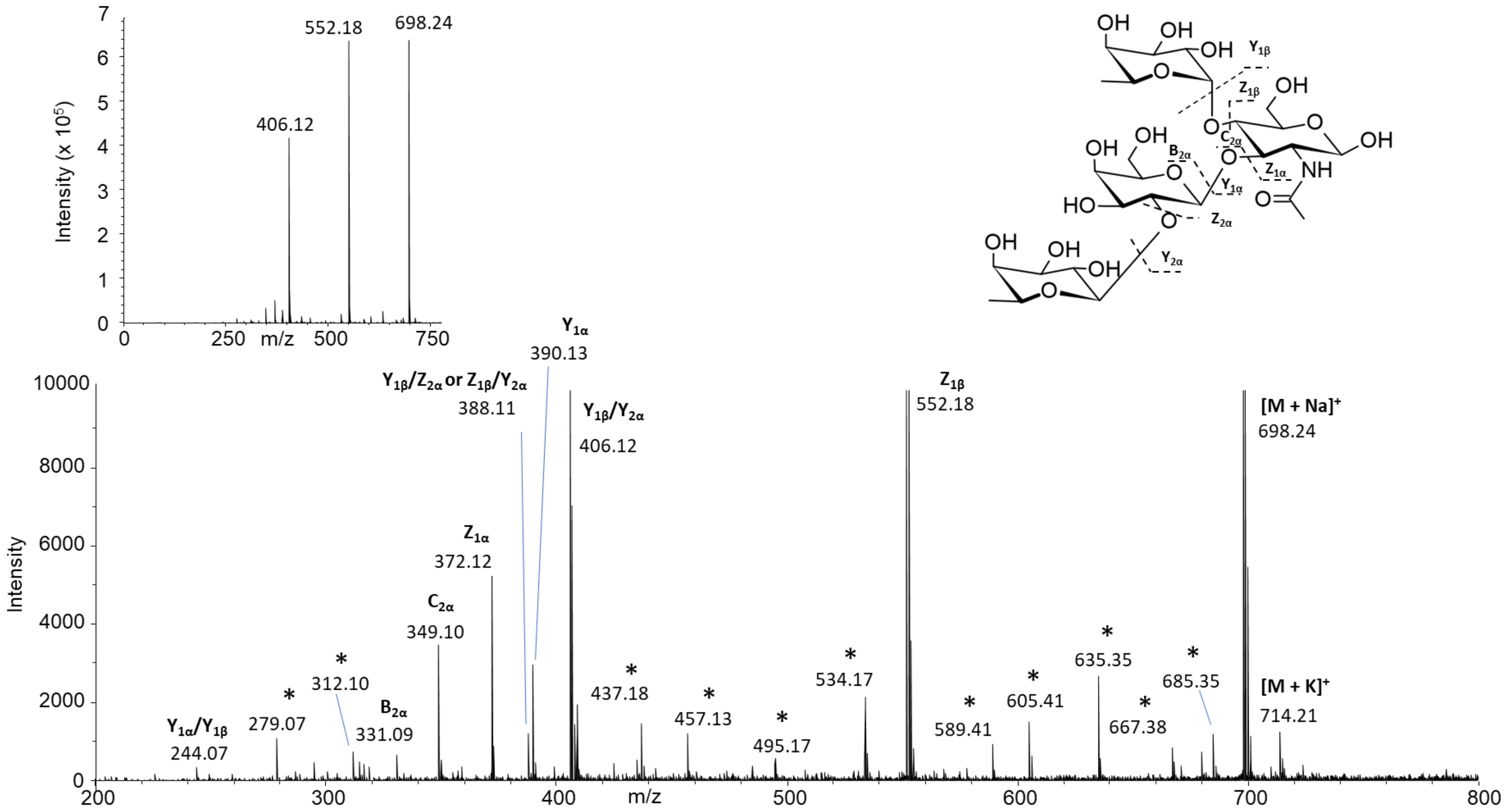

Figure S.45: MS2 (CID) Spectrum - Lewis B Band 2 [M + Na]+

Table S.8: Observed Fragments in MS2 (CID) Spectrum Lewis B Tetrasaccharide [M + Na]+ IMS 2

Type	m/z	Ions
$Y_{1\alpha}Y_{1\beta}$	244.08	Na
$^{0,4}X_{GlcNAc}Z_{1\alpha}$	312.11	Na
$Z_{1\alpha}{}^{1,3}X_{Fuc}$	312.11	Na
$Z_{1\alpha}{}^{2,4}X_{Fuc}$	312.11	Na
$B_{2\alpha}$	331.10	Na
$C_{2\alpha}$	349.11	Na
$Z_{1\alpha}$	372.13	Na
$Y_{1\beta}Z_{2\alpha}$	388.12	Na
$Z_{1\beta}Y_{2\alpha}$	388.12	Na
$Y_{1\alpha}$	390.14	Na
$Y_{1\beta}Y_{2\alpha}$	406.13	Na
$Z_{2\alpha}$	534.18	Na
$Y_{2\alpha}$	552.19	Na
$[M+Na-H_2O]^+$	680.24	Na
$[M+Na]^+$	698.25	Na
